# Cross-linked PVA/PSSA-CNTs based polyelectrolyte membranes with enhanced proton conductivity for fuel cell applications

**DOI:** 10.1038/s41598-026-43521-9

**Published:** 2026-03-27

**Authors:** Eman A. El-Desouky, Emad A. Soliman, Ali A. El-Bardan, Ahmed A. Kassem, Ahmed F. Elerian, M. A. Abu-Saied

**Affiliations:** 1 Chemistry Department, Faculty of Science, Alexandria University, P.O. Box 426, Ibrahimia, 21321 Alexandria Egypt; 2 Polymeric Materials Research Department, Advanced Technology and New Materials Research Institute, City of Scientific Research and Technological Applications (SRTA-CITY), New Borg El-Arab City, Alexandria, 21934 Egypt; 3 Department of Chemistry, Faculty of Science, Al-Azhar University, Cairo, Nasr City, 11884 Egypt; 4 Faculty of Industrial and Energy Technology, Borg EL-Arab Technological University, New Borg El-Arab City, Alexandria Egypt

**Keywords:** PVA, PSSA@CNTs, Proton-exchange membrane (PEM), Chemical crosslinking, Methanol permeability, Proton conductivity, Chemistry, Energy science and technology, Materials science, Nanoscience and technology

## Abstract

Developing polyelectrolyte membranes (PEMs) that possess optimal mechanical and electrochemical properties while maintaining economic efficiency for given applications is challenging. In this research, conductive Polystyrene sulfonic acid (PSSA) interacted with multi-walled carbon nanotubes symbolized (PSSA@CNTs) and incorporated into cross-linked Polyvinyl alcohol (PVA) to create a unique polyelectrolyte membrane. The solution casting procedure was used to prepare cross-linked PVA membranes using varying molar ratios of (PSSA@CNTs). The interaction of cross-linked PVA with PSSA@CNTs was established using different techniques. FT-IR spectroscopy revealed the band at 1720 cm^− 1^, which is the characteristic band of the esterification reaction between the polymer hydroxyl and carbonyl group of the crosslinker Succinic Acid (SA). The band intensity of C = O at nearly 1700 cm^− 1^ increased by increasing the PSSA@CNTs molar ratio. In addition, composite membranes had better swelling resistance, which can be confirmed by water contact angle and water uptake measurements. Transmission electron microscopy (TEM) and scanning electron microscopy (SEM) of surface topography reveal pores on the surface of cross-linked composite membranes, which do not extend through the membrane. This may explain the efficient proton conduction while minimizing methanol crossover. Uniform PSSA@CNTs dispersion in PVA enhanced the mechanical, proton conductivity, and thermal characteristics, which decreased the methanol crossover through the membrane compared to the pristine PVA membrane. The mechanical properties of the prepared membranes by adding PSSA@CNTs are found to improve tensile strength significantly by about 60%. Thermal stability improvements in the composite-prepared membranes were demonstrated through thermogravimetric analysis (TGA). Therefore, PVA/1%PSSA@CNTs-SA membranes showed more desirable properties as a polyelectrolyte membrane with (3.03 meq/g) ion exchange capacity (IEC), water uptake value of (45.11%), and proton conductivity (6.12 × 10^− 2^ S.cm^− 1^). Therefore, the produced membranes exhibit self-extinguish ability and high efficacy with admissible conductivity for electrochemical applications.

## Introduction

Recent advances in energy research have focused on renewable energy, along with the reduction of greenhouse gases such as carbon dioxide, which negatively affect the environment^1–3^. Fuel cells are presently considered a viable, clean energy generation technology due to their high energy conversion efficiency and zero hazardous emissions^4–6^. This applies to various applications in all types of home energy supply, vehicles, laptops, cellular phones, power stations, etc., owing to its minimal emission, simple structure, and excellent efficiency. One of the most effective sustainable alternatives to produce conventional energy is fuel cell technology^7–9^.

There are diverse types of fuel cells, for instance molten carbonate fuel cells (MCFCs), alkaline fuel cells (AFCs), proton exchange membrane fuel cells (PEMFCs), solid oxide fuel cells (SOFCs), and direct methanol fuel cells (DMFCs). Recently, direct methanol fuel cells (DMFC) represent alternatives to traditional electricity sources and are regarded as the most technologically advanced electricity sources among the other fuel cell (FCs) types because of their unique features, such as high fuel energy density, easy liquid fuel storage, low operating temperature, high energy conversion efficiency, simplified system construction, and low pollutant emission.

Conversely, one of the most significant obstacles of DMFCs is the potential for methanol leakage across the polymer electrolyte membrane, along with the challenges of obtaining highly reactive polymer electrolytes with optimal hydration management. Additionally, membrane contamination is a major issue, reducing efficiency by clogging proton-binding sites, particularly due to fuel impurities, biological byproducts, or chemical reactions. To maintain operational efficiency, contaminated polymer membranes must either be repaired or replaced. In addition, the High cost-effective of the key components of fuel cell, mainly electrodes and electrolytes, remains a major obstruction to the well-known implementation of fuel cells. It is noteworthy that the polymer electrolyte membrane (PEM) is a key component in the operation of direct methanol fuel cells (DMFCs), which plays a dual role as it acts as an electrolyte, allowing protons to be supplied from the anode to the cathode, in addition to preventing fuel flow and maintaining structural integrity under operating conditions. The efficiency, robustness, high proton delivery capacity, and cost-effectiveness of DMFCs depend heavily on the properties of these membranes. Therefore, the requirements for these membranes to be suitable for DMFC include high oxidative stability, proton conductivity, good mechanical stability, electrochemical performance in both wet and dry conditions, and low fuel permeability under fuel cell operating conditions^10–12^.

It is widely recognized that ion exchange membranes (IEMs) are a broad class of membranes containing fixed charges in their polymer matrix and Proton exchange membranes (PEMs), are considered as a subclass of ion exchange membranes, are specifically designed for proton transfer while minimizing the transport of fuel molecules such as methanol. Anion exchange membranes (AEMs), with exchangeable anions and fixed cation groups (such as PO4³⁻, Cl⁻, CO3²⁻, and SO4²⁻), and cation exchange membranes (CEMs), with exchangeable cations (such as Ca²⁺, Na⁺, and K⁺), are the two main types of ion exchange membranes. Sulfone groups are preferred in the design of cation exchange membranes due to their high ion exchange capacity, water adsorption, and ionic conductivity^13–15^.

Additionally, the amount of water absorbed and the movement of ions through the membrane are directly correlated in IEMs^16,17^. Historically, CEMs have made greater strides than AEMs, mainly because of their ability to achieve high H^+^ conductivity. Due to their high mechanical, chemical, and ionic conductivity properties, perfluorinated ionic polymers with sulfonate groups (-SO_3_^−1^) and pairs of monovalent ions, like Nafion, are suitable for use as membranes^18–20^. However, these membranes’ cost, high fuel crossover, and complexity limited their use in commercial and laboratory research. As a result, numerous studies are being carried out to develop a cost-effective PEM as a replacement for Nafion membrane in order to solve these issues^21,22^. Numerous publications on the development of PEMs from perfluorinated membranes modified with polybenzimidazoles, polyimides, and poly (arylene ether sulfone), polyvinyl chloride (PVC)^23,24^, chitosan (CS)^25,26^, poly (vinyl alcohol) (PVA), and poly (ether ether ketone) (PEEK) as PEMs.

Polyvinyl alcohol (PVA) is a chemically stable polymer with methanol tolerance characteristics and good film-forming capabilities^27^. PVA is a semicrystalline and linear polymer comprised of a backbone carbon chain and a functional hydroxyl group (OH). PVA-based membranes are gaining popularity due to their biocompatibility, flexibility, and biodegradability. Particularly, PVA has received a lot of interest because of its excellent mechanical properties, biocompatibility, nontoxicity, hydrophilicity, and good film-forming ability. Nevertheless, mechanical characteristics degrade significantly when wet, and it has poor cell adherence. Various strategies have been investigated to address these restrictions^28–30^. Prior investigations have utilized a range of chemical crosslinkers to enhance the physicochemical and mechanical properties of PVA-based membranes, including glutaraldehyde (Glu)^31^, Sodium hydroxide^32^, and other multifunctional agents^33,34^. These crosslinkers were primarily introduced to improve dimensional stability, reduce excessive swelling, and enhance proton transport behavior. However, limitations related to stability, environmental impact, or performance trade-offs remain. Therefore, this research focuses on the rational design of polymeric membranes, the incorporation of suitable crosslinking agents, and the control modification of their physicochemical properties. Recent advancements in the properties of PEMs have been achieved by incorporating multi-walled carbon nanotubes (CNTs) as reinforcing fillers. The incorporation of CNTs within polymer matrices significantly enhances the mechanical properties of polymer/CNTs nanocomposites. However, pristine CNTs tend to aggregate into bundles or ropes due to strong van der Waals interactions, which hinder their uniform dispersion in the polymer matrix, thereby limiting their processability and nanoscale performance^35,36^. To address this challenge, surface functionalization of CNTs through carboxylation and sulfonation has been employed to improve their dispersion and compatibility within the polymer matrix, enabling their effective utilization in various applications. Additionally, the incorporation of polystyrene sulfonic acid (PSSA) onto the CNTs surface further enhances dispersion and introduces proton-conducting sulfonic acid groups, thereby improving the membrane’s ionic conductivity^37,38^. While previous studies have focused on PVA-based proton-exchange membranes and carbon nanotube (CNT)-reinforced polymers, the majority of studies have primarily focused on improving individual membrane properties rather than achieving a systematic balance between proton conductivity and methanol permeability, which is crucial for direct methanol fuel cell (DMFC) applications. Notably, the synergistic effect of surface-functionalized CNT incorporation combined with tunable organic crosslinking within a single membrane platform has remained largely unexplored^18,19^.

This work details the fabrication of polyelectrolyte membrane based on cross-linked PVA/PSSA@CNTs. Carbon nanotubes (CNTs) were initially functionalized at the surface through acid treatment to facilitate stronger interfacial adhesion between CNTs and the polymer matrix, then after that poly(styrene sulfonic acid) (PSSA) was inserted to introduce additional proton-conducting functionalities. After that, these functionalized PSSA@CNTs were then integrated into a PVA polymer matrix, which functioned as the core of the membrane system. In addition, to enhance the membrane network structure, different chemical crosslinkers, including citric acid (CA), succinic acid (SA), and their combination (CA/SA, 1:1), were employed to enhance the properties of developed polyelectrolytic membranes. The employment of citric acid (CA) as a chemical crosslinking agent assists the efficient realization of ester bonds with the hydroxyl groups in polyvinyl alcohol (PVA), because of its multifunctional carboxyl groups. This improves structural integrity and proton transport efficiency, as well as contributing to its environmentally friendly properties. Succinic acid (SA), used individually or in combination with citric acid, helps control the crosslinking density while providing proton-donating functionalities. These chemical bonding agents were used individually (CA or SA) and in a 1:1 ratio (CA: SA), allowing for precise control of the membrane network structure. This approach enables a balanced enhancement of membrane performance, including dimensional stability, electrochemical properties, and long-term stability, making it well-suited for direct methanol fuel cell (DMFC) applications. This approach allowed precise control over crosslink density and network morphology, resulting in the preparation of PVA/PSSA@CNTs-CA, PVA/PSSA@CNTs-SA, and PVA/PSSA@CNTs-SC membranes. Communally, this integrated strategy was designed to achieve a harmonious improvement in proton conductivity, methanol barrier properties, dimensional stability, and mechanical strength within a single membrane platform. Comprehensive characterization of the prepared polyelectrolyte membranes was analyzed using various characterization technique, including scanning electron microscopy (SEM), CHNS elemental analysis, energy-dispersive X-ray spectroscopy (EDX), Raman spectroscopy, thermogravimetric analysis (TGA), Fourier-transform infrared spectroscopy (FTIR), and transmission electron microscopy (TEM). Simultaneously, essential membrane properties were comprehensively assessed, including proton conductivity, ion exchange capacity (IEC), water uptake, contact angle, dimensional stability (in-plane, through-plane), mechanical strength, and methanol permeability. Importantly, to the best of our knowledge, such a systematic investigation combining PSSA-functionalized CNTs with dual organic crosslinking to address the conductivity–permeability trade-off in PVA-based PEMs has not been reported before. As a result, this study offers valuable insights into the structure–property relationships that govern the performance of cost-effective PEMs, providing a promising direction for DMFC applications.

## Materials and methodology

### Materials

PVA from Across Organics USA, with Hydrolyzed percentage (95.5–96.5%) and (85,000–124,000) average M. wt., Ethanol was obtained from Doummar & Sons Company, Germany. Sodium Hydroxide pellets (98% purity) were obtained from Central Drug House Ltd. (New Delhi, India). Sulfuric acid (99%) from United Co., Egypt. (Citric acid-CA, 99%), (Succinic acid-SA, 99%) purchased from Sigma-Aldrich USA Company. Multi-walled carbon nanotubes (CNTs) from SRL chemicals India with 10–30 μm length, Poly (Poly (sodium 4-styrene sulfonate) (PSS) (~ 70,000average Mw) was obtained from Across Organics USA. Hydrochloric acid (36% purity) and Nitric acid (65%) from Sigma-Aldrich USA Company. All chemicals, without additional purification, were utilized as obtained.

### Synthesis of PSSA@CNTs

**Fig. 1:**
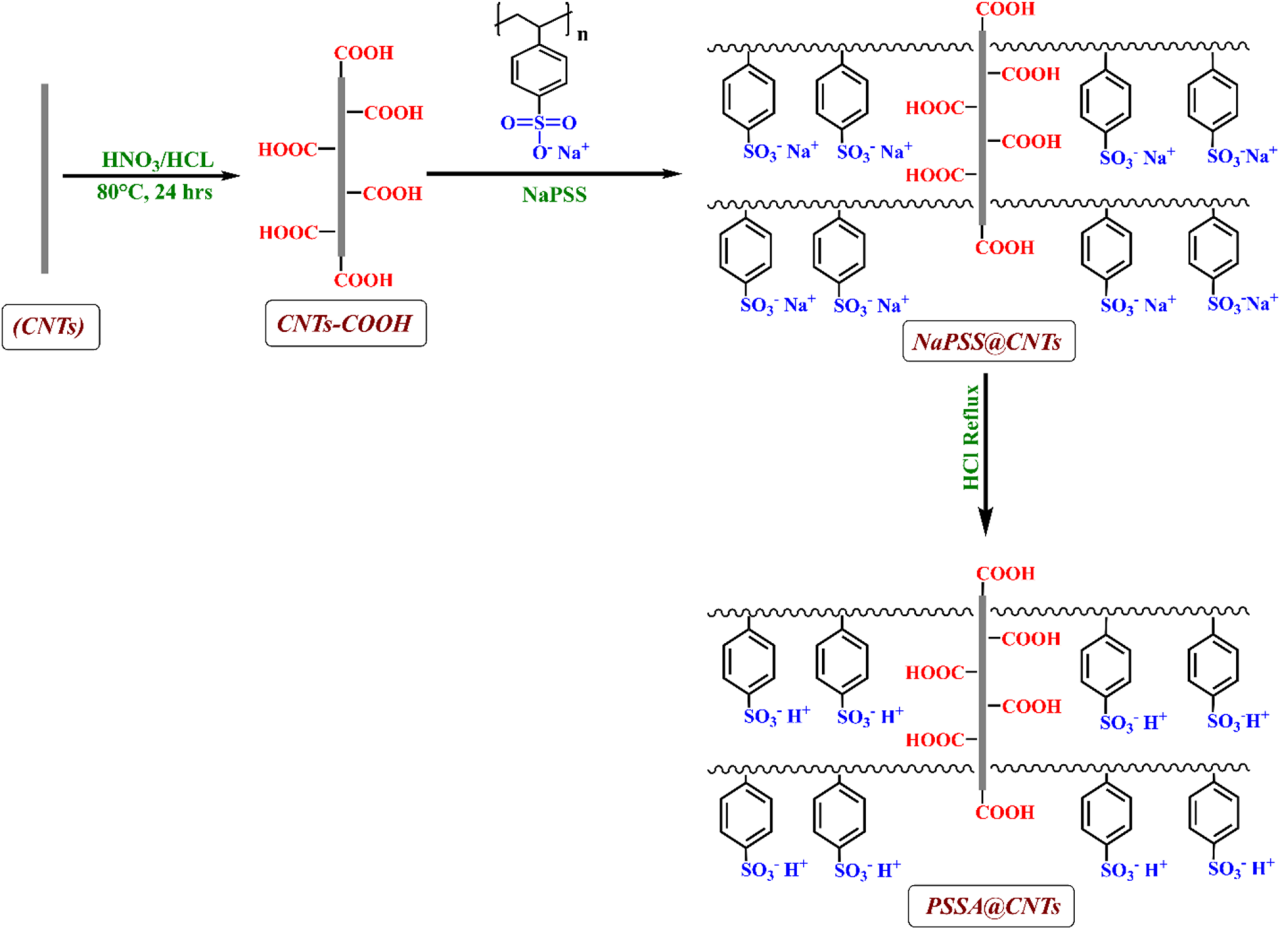
Surface Modification of CNTs via PSSA incorporation 39^41^.

Carbon nanotubes (CNTs) were initially sonicated for 30 min in a 1:1 mixture of concentrated nitric acid (65%) and hydrochloric acid (37%) to disperse them. Afterward, it was repeatedly washed and filtered to remove any remaining acid on the surface and finally dried under vacuum for 24 h. at 80 °C ^39–41^. Notably, this acid treatment is well established for introducing carboxyl (-COOH) groups predominantly at defect sites and tube ends of CNTs, thereby enhancing their surface reactivity and compatibility with subsequent polymer functionalization. This treatment introduced predominantly carboxyl groups (-COOH) onto the CNT surfaces. The obtained CNTs were then coated with (3 wt%) conductive poly (sodium-4-styrene sulfonate) (NaPSS) solution and stirred for 3 h. at 80 °C. The collected sulfonated CNTs were filtered, and the product was repeatedly washed and filtered until neutral pH, then dried for 24 h. in a vacuum. Finally, the obtained NaPSS@CNTs were hydrolyzed with dilute HCl to produce PSSA@CNTs with (-SO_3_H) functional group (Fig. 1).

### Preparation of cross-linked PVA/PSSA@CNTs-based PEMs

Cross-linked PVA/PSSA@CNTs membranes were synthesized using the solution-casting method. Initially, a pre-weighed quantity of PVA was dissolved in deionized water for 5 h. at 90 °C with magnetic stirring to generate a homogeneous PVA polymer solution (6 wt%). For functionalization, various amounts of PSSA@CNTs (0.25, 0.5, and 1 wt%) were dispersed into PVA viscous solutions and sonicated for 1 h. to achieve a homogeneous mixture.

The cross-linking approach was used to improve the durability of the resulting polymer composite membranes. Chemical cross-linkers such as Citric acid (CA), Succinic acid (SA), and a combination of Citric acid and Succinic acid (CA/SA 1:1) (denoted as SC) were individually introduced into PVA/PSSA@CNTs solutions and stirred overnight to generate a restricted chemical network via PVA/PSSA@CNTs chain cross-linking. Subsequently, the homogeneous solutions were spread onto a plastic plate and allowed to dry overnight at 40 °C.

Eventually, the developed membranes were immersed in a 2 M sulfuric acid (H_2_SO_4_) solution to ensure complete protonation of the sulfonic acid (-SO_3_H) groups and to enhance proton transport, and were respectively coded with the following symbols: PVA/PSSA@CNTs-CA, PVA/PSSA@CNTs-SA, and PVA/PSSA@CNTs-SC.

**Fig. 2:**
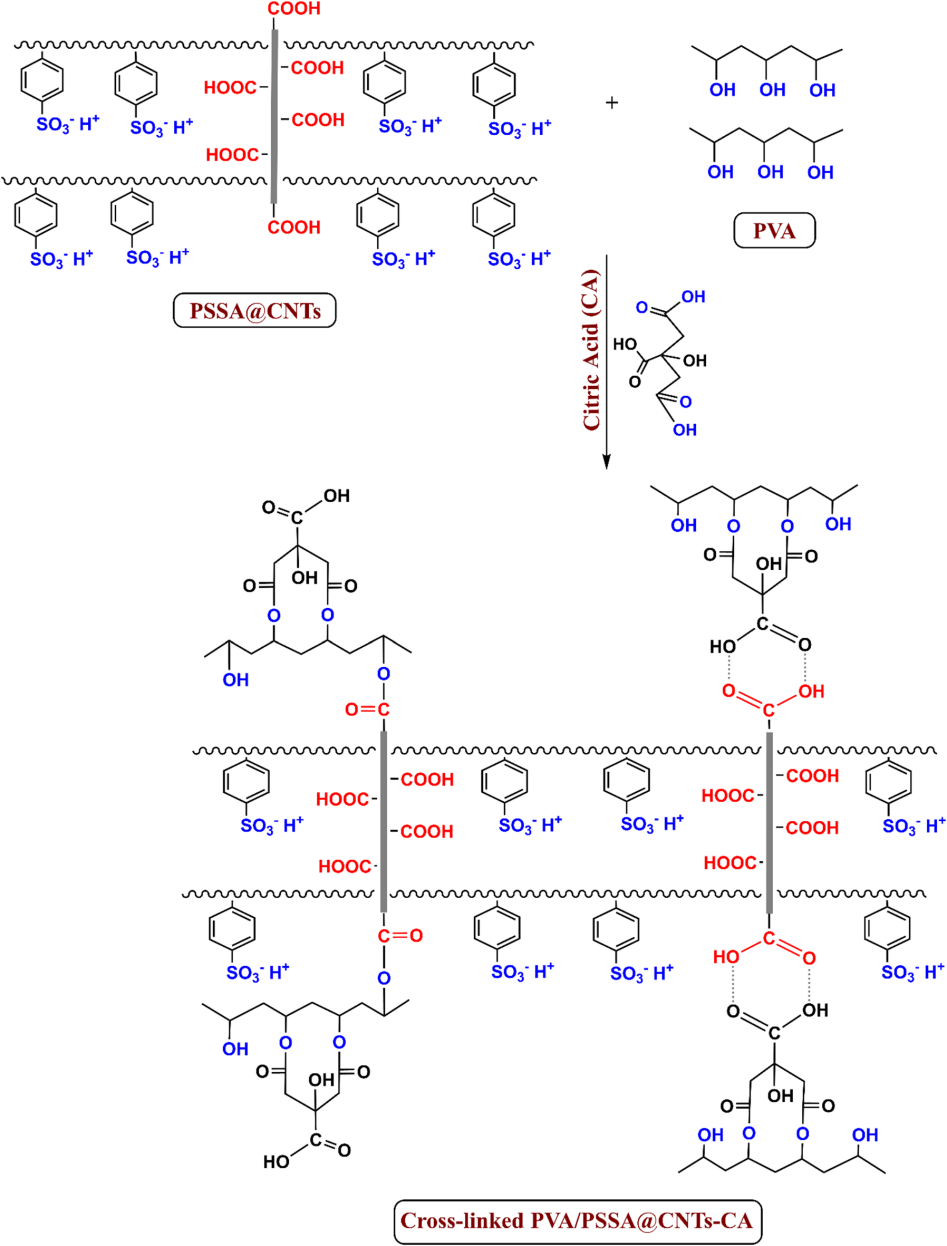
Proposed interaction mechanism of PVA/PSSA@CNTs with Citric Acid (CA).

**Fig. 3:**
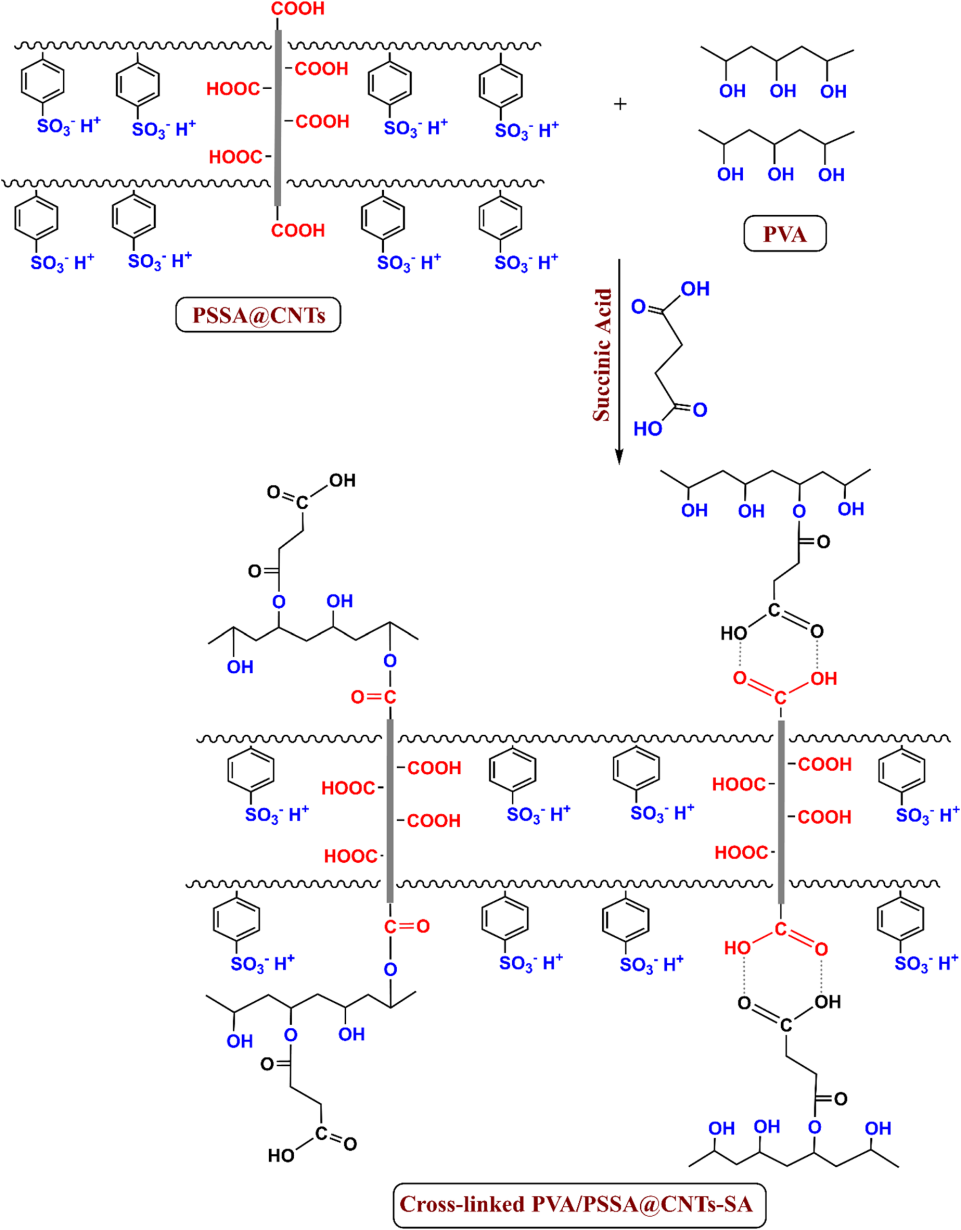
Proposed interaction mechanism of PVA/PSSA@CNTs with Succinic Acid (SA).

The proposed reaction mechanisms, including the interactions between PSSA@CNTs and PVA as well as their crosslinking with CA and SA, are illustrated in Figs. 2 and 3. Citric acid (CA) and succinic acid (SA) act as multifunctional crosslinkers, forming ester bonds with hydroxyl groups in PVA and potentially reacting with functional groups on PSSA-modified carbon nanotubes (CNT–COOH groups) through physical interactions, particularly hydrogen bonds forming a cyclic dimer. Additionally, chemical crosslinking may occur between the carboxyl groups in the crosslinker and the hydroxyl groups in PVA forming ester linkage, enhancing the structural integrity of the membrane^42–44^.

### Membrane characterization

The morphology of prepared membranes was investigated by TEM (JEOL Ltd., JSM-5300, Japan), accelerated with 200 kV. In addition, SEM (JEOL JSM-6360LV, Japan) was used to characterize the morphological characteristics of the prepared membranes^45–47^.

The membranes’ thermal properties were inspected under N_2_ gas pressure and heating rate 20 °C /min, using a Shimadzu TGA-50 spectrometer^48,49^.

The mechanical properties of the prepared CA-PVA/SSCA-based membranes were assessed using a UTM, Shimadzu, Kyoto, Japan, model AG-I, 5KN universal tensile testing machine, using a stretching rate of 5 mm/min^50,51^.

The chemical interactions were studied by FT-IR (Shimadzu-Japan, FT-IR-8400 S) and Raman spectrum (SENTERRA-Bruker, Germany)^52–54^.

Elemental analysis (EA) was obtained with the Vario Micro Cube Elementar CHNS Analyzer- Germany. The dry combustion method determined the total carbon, hydrogen, Sulfur, and nitrogen^55^.

Swelling behavior is also studied for prepared membranes. The water uptake percentage (WU%) was measured methods at room temperature by gravimetric measuring. The 1 cm × 1 cm membrane strips were fully dehydrated in an oven, and the obtained dried samples were immediately weighed (Wd). The samples were then submerged in 20 ml of D.I water for 24 h. and then the wet sample (Ww) was weighed, and WU% was calculated by using Eq. (1). Additionally, the same method was used to determine Methanol uptake (MU%) by using Methanol solvent instead of D.I water^56,57^.

1 $$\mathbf{W}\mathbf{U}\left(\mathbf{\%}\right)=\frac{(\mathbf{W}\mathbf{w}-\mathbf{W}\mathbf{d})}{\mathbf{W}\mathbf{d}}\times100$$

2 $$\mathbf{M}\mathbf{U}\left(\mathbf{\%}\right)=\frac{(\mathbf{W}\mathbf{w}-\mathbf{W}\mathbf{d})}{\mathbf{W}\mathbf{d}}\times100$$

*Wd* and *Ww* are the membrane’s average dry weight and wet weight (after 24 h. immersion), respectively.

Dimensional swelling (In-Plane and Through-Plane) was evaluated by using Eqs. (3) & (4) to assess the structural stability of the membranes under hydrated conditions. Membrane dimensions were measured in the dry state and after equilibration in deionized water at the specified temperature. The swelling ratios were calculated as follows:

3 $$\mathrm{Δ}\mathrm{A}\text{}\mathrm{(\%)}=\frac{{\boldsymbol{L}}_{\boldsymbol{w}\boldsymbol{e}\boldsymbol{t}}-{\boldsymbol{L}}_{\boldsymbol{d}\boldsymbol{r}\boldsymbol{y}}}{{\boldsymbol{L}}_{\boldsymbol{d}\boldsymbol{r}\boldsymbol{y}}}\times100$$

4 $$\mathrm{Δ}\mathrm{T\:(\%)}=\frac{{\boldsymbol{T}}_{\boldsymbol{w}\boldsymbol{e}\boldsymbol{t}}-{\boldsymbol{T}}_{\boldsymbol{d}\boldsymbol{r}\boldsymbol{y}}}{{\boldsymbol{T}}_{\boldsymbol{d}\boldsymbol{r}\boldsymbol{y}}}\times100$$

Where **Lwet** and **L**dry represent the length (or width) of the wet membrane and the length (or width) of the dry membrane, respectively, while **Twet** and **Tdry** represent the thickness of the wet membrane and the thickness of the dry membrane, respectively.

The gel fraction (%) of the membranes, as an indicator of crosslinking density, was evaluated. Membrane samples were first dried at 40 °C for 24 h and weighed accurately using a four-decimal electronic analytical balance (**W**_**0**_). To remove un-crosslinked fractions, the dried samples were immersed in deionized water for 24 h and then dried in a vacuum oven at 40 °C to constant weight (**W**_**1**_)^58^. All measurements were performed in triplicate. The gel fraction was calculated according to the following equation:

5 $$\mathrm{Gel\:fraction\:\:(\%)}=\frac{{\boldsymbol{W}}_{1}}{{\boldsymbol{W}}_{0}}\times100$$

The Water contact angle (WCA) was determined by using (ramé-hart) instrument to obtain the wettability degree of the prepared membranes^59^.

Furthermore, the membranes’ ion-exchange capacity (IEC) was determined. After being in contact with 0.1 M HCl aqueous solution for 24 h. the membranes underwent three rounds of deionized water washing. IEC was calculated using Eq. (6) by using the titration method and phenolphthalein as an indicator with a 0.02 M NaOH solution^60–62^.

6 $$\mathbf{I}\mathbf{E}\mathbf{C}=\frac{\mathbf{M}\times{\boldsymbol{V}}_{\boldsymbol{N}\boldsymbol{a}\boldsymbol{O}\boldsymbol{H}}}{\mathbf{W}\mathbf{d}}$$

Where M is the molarity of the NaOH, Wd is the weight of the dried membranes, and V is the volume consumed for each titration of NaOH solution.

Oxidation stability, which reflected the lifetime of the synthesized membranes, was investigated through the Fenton test. The dried membrane specimens (1 × 4 cm^2^) were soaked in Fenton’s reagent (2 ppm FeSO_4_ in 3% H_2_O_2_) for 1 h. 80 °C, and pH = 3. Equation (7) calculated the oxidative stability based on the changing of weight before and after immersion between specimens^63,64^.

7 $$\mathbf{O}\mathbf{x}\mathbf{i}\mathbf{d}\mathbf{a}\mathbf{t}\mathbf{i}\mathbf{v}\mathbf{e}\mathbf{s}\mathbf{t}\mathbf{a}\mathbf{b}\mathbf{i}\mathbf{l}\mathbf{i}\mathbf{t}\mathbf{y}\left(\mathbf{\%}\right)=\frac{{(\boldsymbol{W}}_{0}-{\boldsymbol{W}}_{1})}{{\boldsymbol{W}}_{0}}\boldsymbol{X}100$$

W_o_ and W_1_ embody the weight of samples before and after oxidation in grams, respectively.

The methanol permeability (P) of the sample membrane can be determined by using two cells separated by the membrane specimen. One compartment (CP_B_) received 100 mL of deionized water, and the other compartment (CP_A_) received 100 mL of the 1 mol/L methanol solution. During the experiment, the solutions in CP_A_ and CP_B_ were thoroughly mixed, and samples from CP_B_ were taken at regular intervals. The methanol permeability was measured over time based on the change in CH_3_OH concentration. A portable density meter (DMA™ 35) was used to determine the methanol concentration of the sample^65,66^. Methanol permeation (P) was calculated using Eq. (8), as follows:

8 $$\boldsymbol{P}=\frac{\boldsymbol{K}\boldsymbol{V}\boldsymbol{L}}{\mathbf{A}{\mathbf{C}}_{\boldsymbol{A}}}$$

Where C_A_ signifies the methanol concentration found in CP_A_, V is the volume after permeability of the solution in CP_B_ occurs, K is the permeation curve slope, and A and L are the area and thickness of the membrane, respectively.

The proton conductivity of the developed polyelectrolyte membranes was measured by electrochemical impedance spectroscopy (EIS) using a Gamry Instruments Reference 3000 potentiostat/galvanostat. The measurements were carried out at room temperature (~ 25 °C) in the frequency range of 100 Hz to 1 MHz under an AC perturbation amplitude of 10 mV.

A homemade two-electrode cell was employed, consisting of two platinum (Pt) wire electrodes mounted on insulating frames, with the membrane sample sandwiched between the electrode compartments. Before measurement, the membrane samples were cut into rectangular pieces with defined thickness and surface area. The obtained impedance spectra were analyzed using an equivalent circuit model comprising a constant phase element (CPE) in series with membrane resistance (R). The bulk membrane resistance was extracted from the high-frequency intercept of the Nyquist plot. The Eq. (9) is used to calculate the proton conductivity of membranes using impedance data^67,68^.

9 $$\boldsymbol{\upsigma}\left(\mathbf{s}/\mathbf{c}\mathbf{m}\right)=\frac{\mathbf{L}}{\mathbf{R}\mathbf{W}\mathbf{T}}$$

where **L** (cm) is the distance between two electrodes, **σ** (S cm^− 1^) is the Proton conductivity, **R** is the membrane resistance, and **T** and **W** are the membrane thickness (cm) and width (cm), respectively.

To assess the efficiency of polyelectrolyte membranes for employment in DMFCs, the following Eq. (10) was used, which expresses ionic conductivity and methanol permeability.

10 $$\phi=\frac{\boldsymbol{\upsigma}}{P}$$

where (ɸ) denotes the effectiveness of the membrane as a function of the connection between proton conductivity (σ) and methanol permeability (P). This ratio serves as a Figure-of-merit that qualitatively evaluates the balance between proton transport capability and methanol crossover resistance, allowing comparison of different membranes’ overall performance^69,70^.

## Results and discussion

### Morphological features of the prepared PEMs

#### Transmission electron microscope (TEM)

**Fig. 4:**
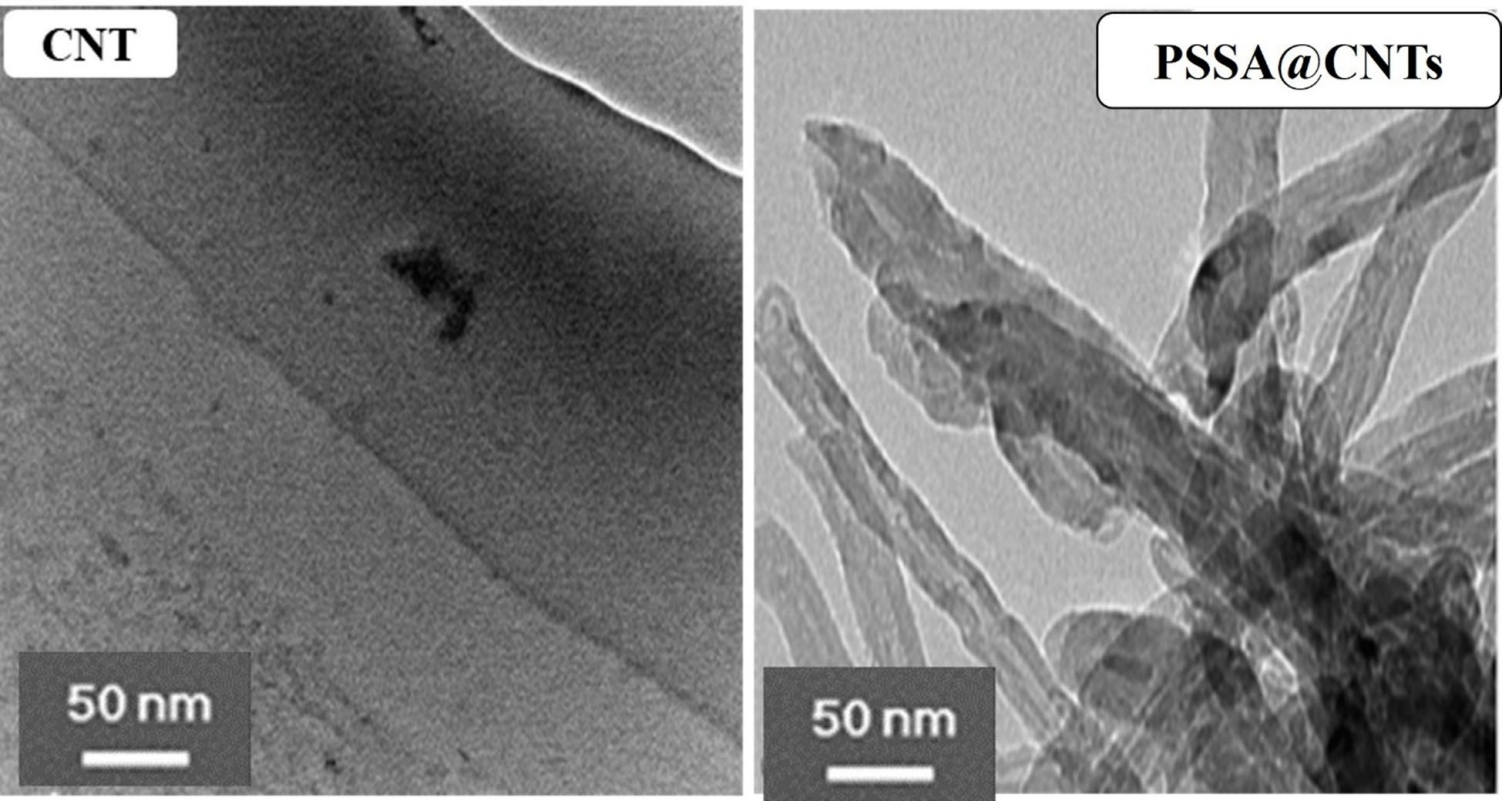
TEM of CNT and PSSA@CNTs.

TEM characterizations of CNTs and (PSSA@CNTs) as shown in Fig. 4 have been carried out to obtain morphological properties of the CNTs before and after alterations. Morphological features of PSSA@CNTs using TEM show that the tubular structure of CNTs was preserved and unaffected by the presence of PSSA. Vander Waals interactions between the tubes keep pristine CNTs together as chains. On the other hand, PSSA-modified CNTs are scattered widely because of the electrostatic repulsion that PSSA’s sulfonic acid groups provide^71^.

**Fig. 5:**
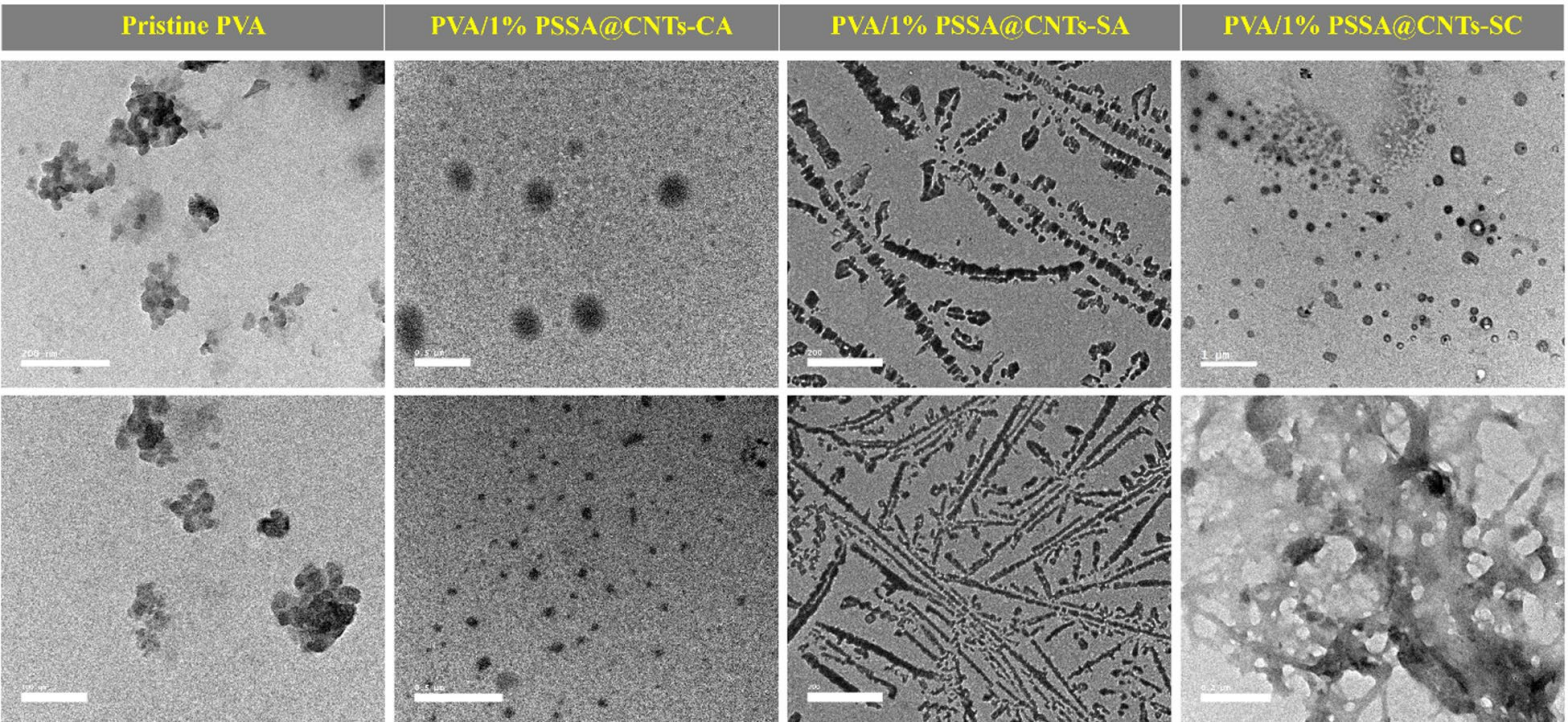
TEM of pristine PVA and cross-linked PVA/PSSA@CNTs-based polyelectrolytic membranes.

Notably, TEM images of the pristine PVA membrane (Fig. 5) show apparent heterogeneous contrast features. These features are primarily attributed to sample preparation defects and variations in electron density contrast during TEM analysis, rather than actual phase separation. PVA is known to be a semi-crystalline polymer; therefore, the small dark areas may indicate localized variations in crystallinity or differences in hydrogen bond density within the polymer matrix. Relevantly, no clear phase border line or detached bands were observed, confirming that the pristine PVA forms a continuous single-phase matrix, whereas the functionalized membranes (PVA/PSSA@CNTs) reveal a clear distribution of the incorporated of PSSA@CNTs within the PVA polymer matrix^72,73^. The particles of PSSA@CNTs appear as dark spots or nanofibers, indicating good dispersion with minimal agglomeration. PVA/PSSA@CNTs-CA showed individual spherical particles, while PVA/PSSA@CNTs-SA membrane images show a lamellar shape. On the other hand, PVA/PSSA@CNTs-SC show a spherical particle, and some have an agglomerated state. Thus, TEM images indicate that PSSA@CNTs and crosslinkers are well distributed within the PVA domains, showing noticeable compatibility between all components. Hence, all PVA cross-linked membranes exhibit good structural integrity within the PVA matrix^74–76^.

**Fig. 6:**
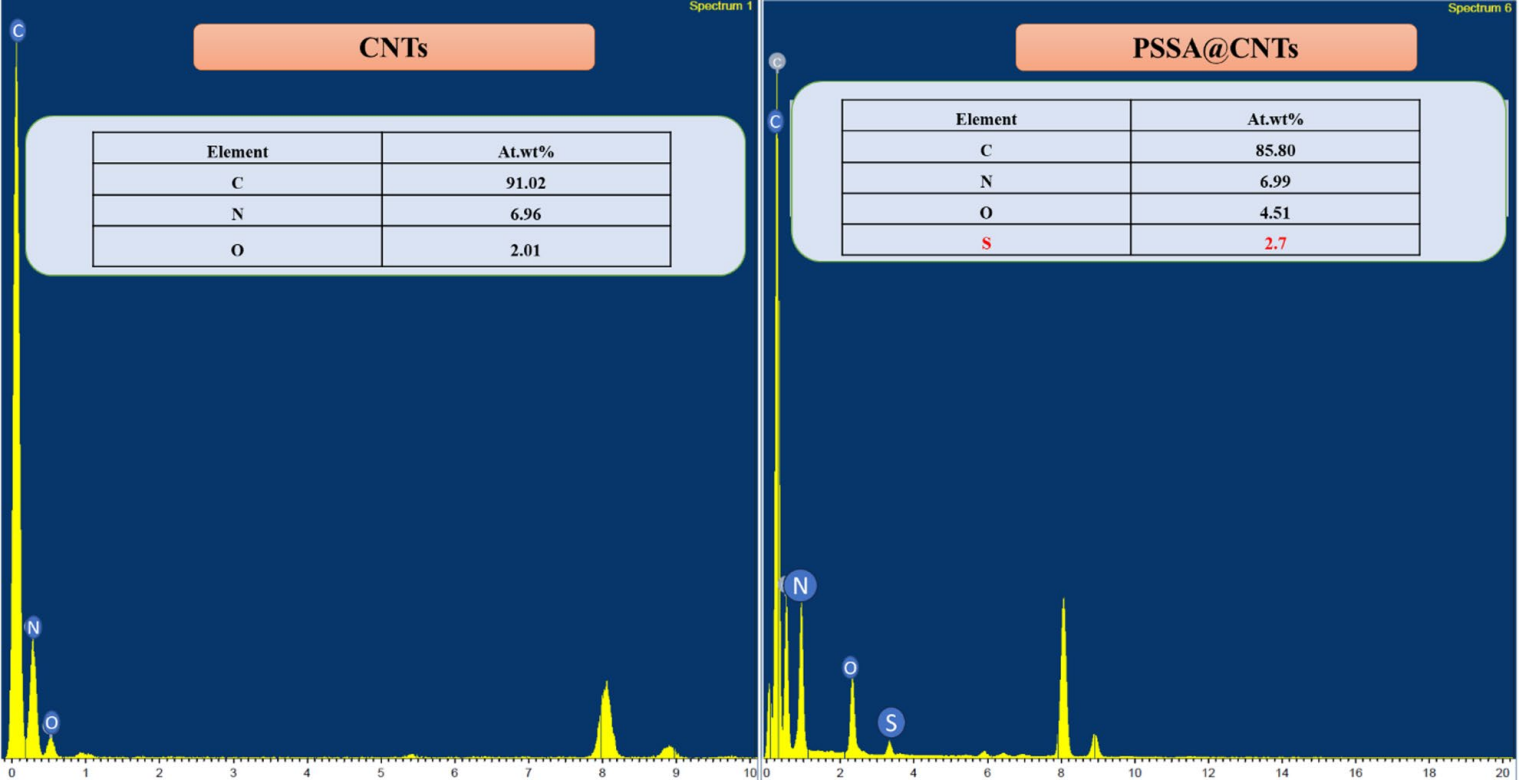
EDX spectra of a pristine CNT and PSSA@CNTs.

Additionally, EDX was used to analyze the elemental profiles of the prepared materials, as it is a microanalysis technique that provides information about the elemental composition of the samples. Figure 6 illustrates the atomic weight percentages for the CNTs and PSSA@CNTs, as shown in the inset through EDAX spectra. The successful modification of CNTs with PSSA is evident from the detection of sulfur (2.70 wt%) from the -SO_3_H group of PSSA, along with higher oxygen content in PSSA@CNTs (4.51 At. wt%) compared to (2.01 At. wt%) of CNTs.

#### Topography and cross-sectional features

**Fig. 7:**
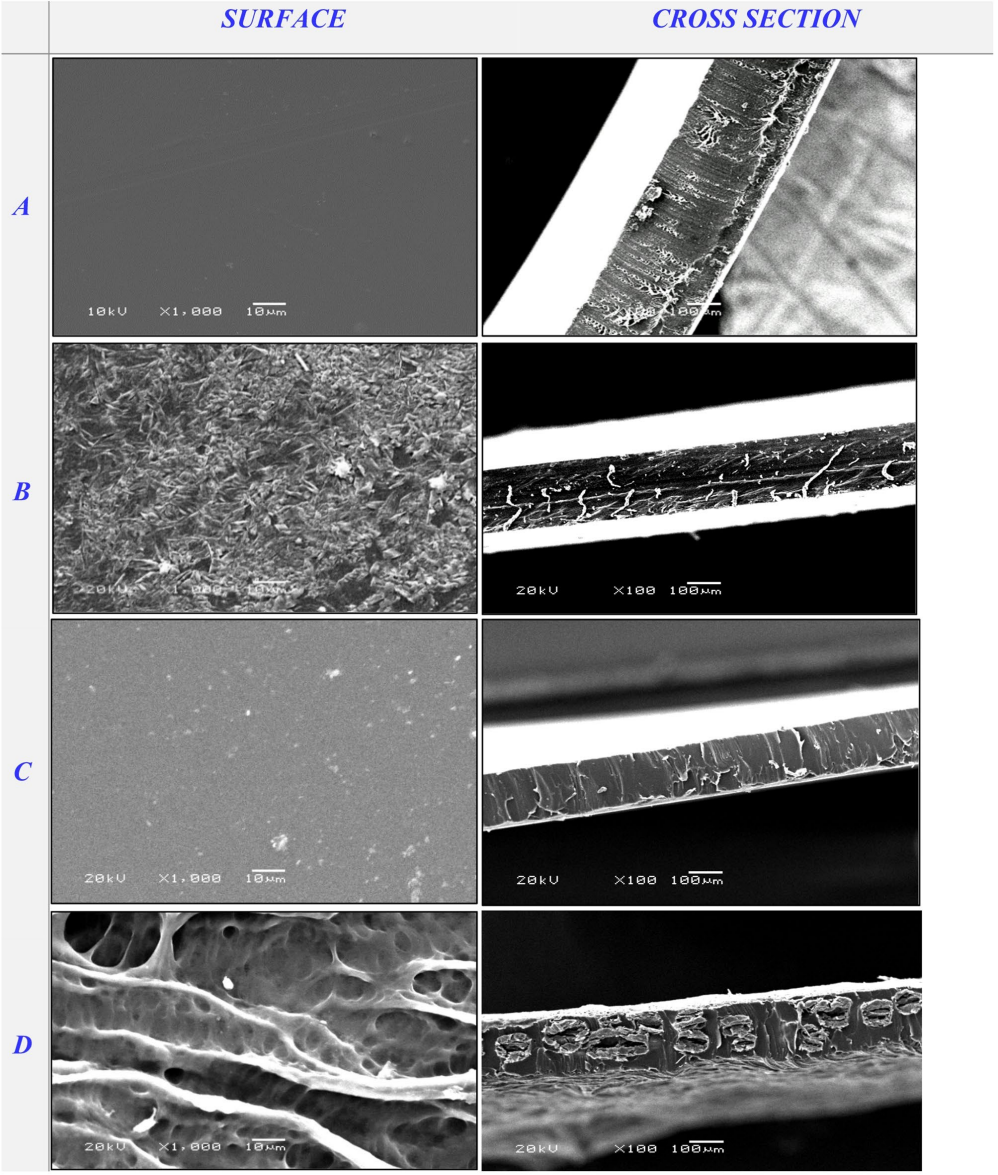
SEM micrograph of the surface and cross-section of (**A**) Pristine PVA, (**B**) PVA/0.25% PSSA@CNTs-SA, (**C**) PVA/0.5% PSSA@CNTs-SA, and (**D**) PVA/1% PSSA@CNTs-SA blend-based membranes.

**Fig. 8:**
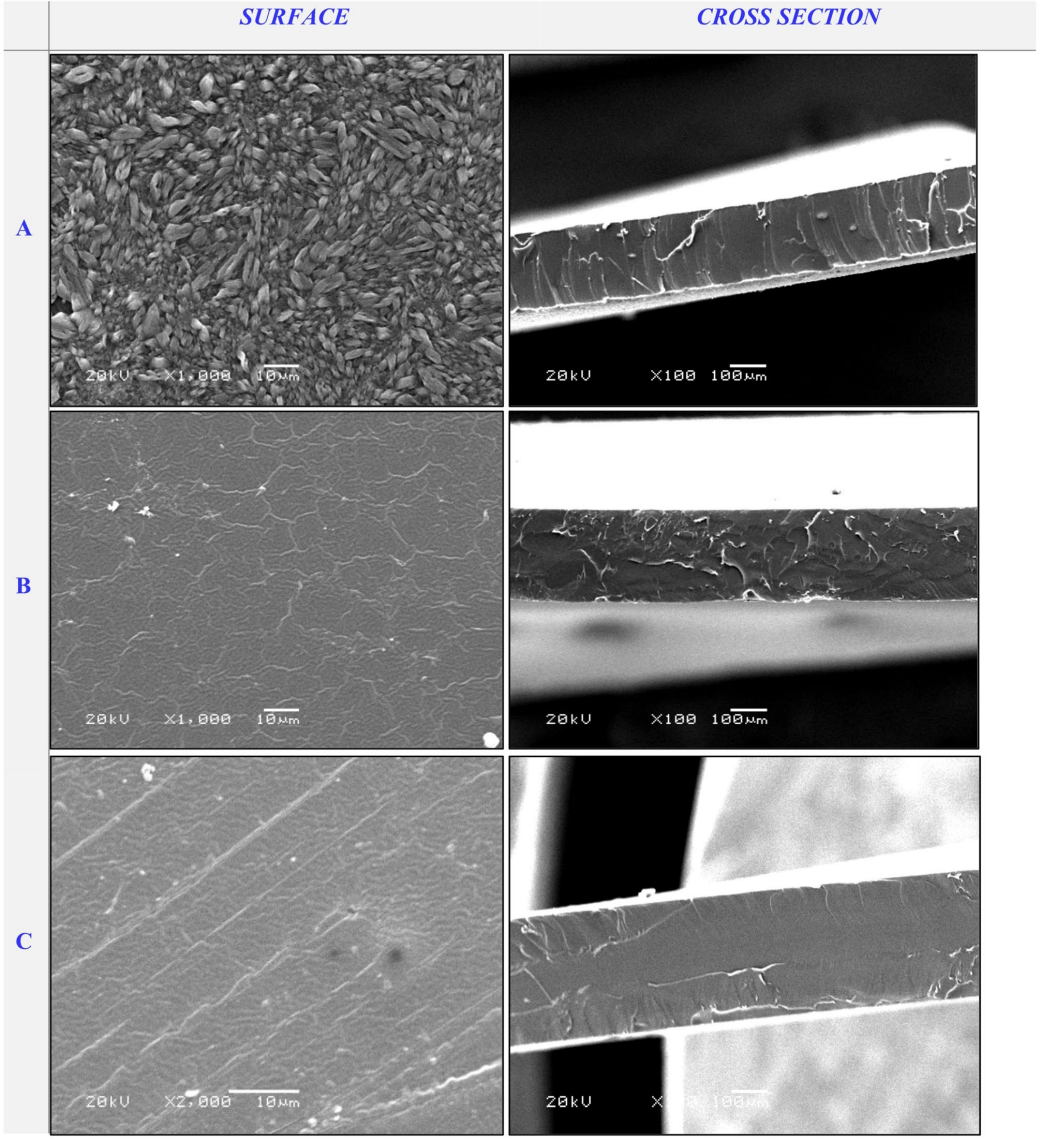
SEM micrograph of the surface and cross-section of (**A**) PVA/0.25% PSSA@CNTs-CA, (**B**) PVA/0.5% PSSA@CNTs-CA, and (**C**) PVA/1% PSSA@CNTs-CA blend-based membranes.

**Fig. 9:**
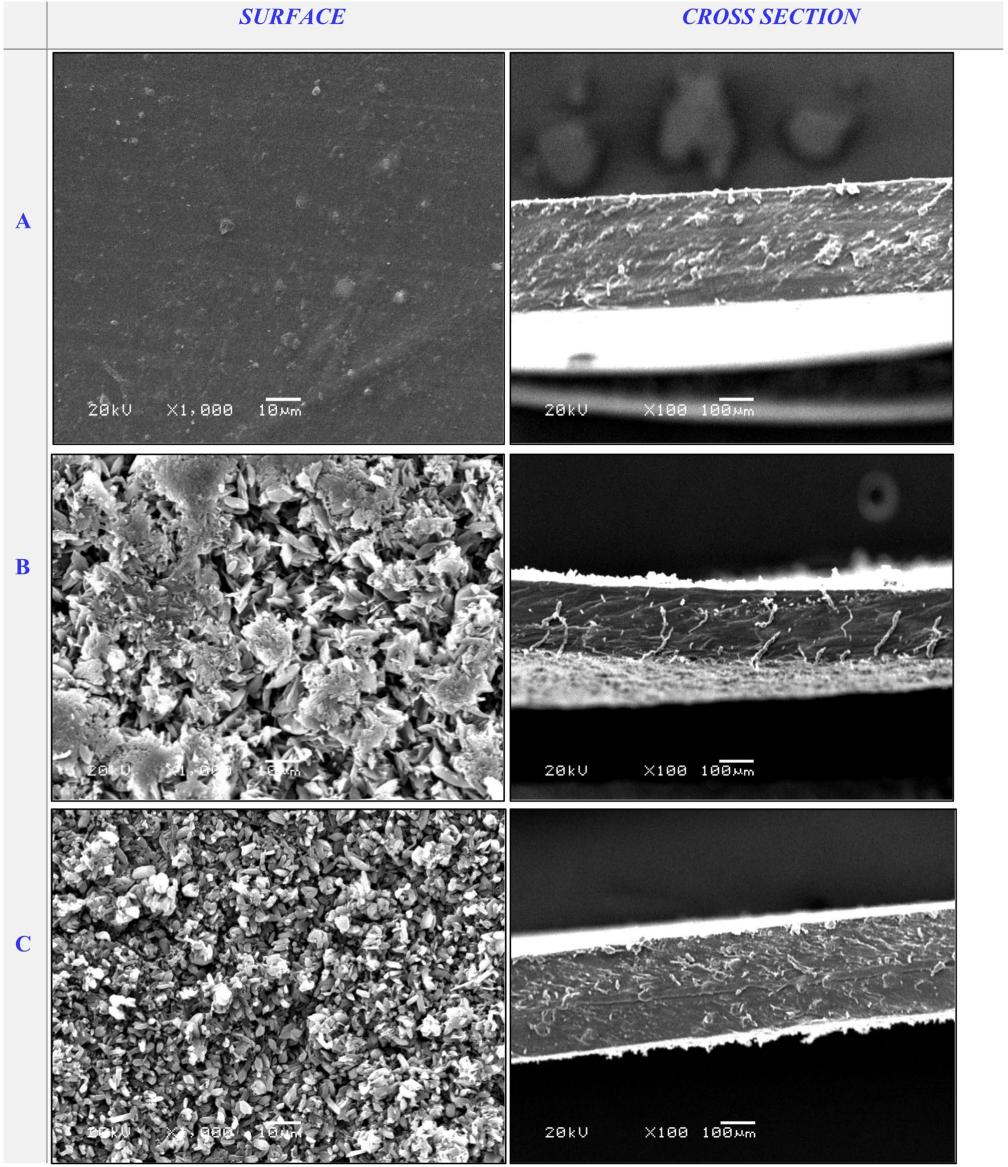
SEM micrograph of the surface and cross-section of (**A**) PVA/0.25% PSSA@CNTs-SC, (**B**) PVA/0.5% PSSA@CNTs-SC, and (**C**) PVA/1% PSSA@CNTs-SC blend-based membranes.

SEM micrographs were utilized to analyze the surface and cross-sectional morphology of cross-linked PVA/PSSA@CNTs membranes compared to pristine PVA. Figure 7a shows typical SEM images of pristine PVA compared to the functionalized polyelectrolyte membranes based on (PVA/PSSA@CNTs-SA) (Fig. 7B-D), (PVA/PSSA@CNTs-CA) (Fig. 8), and (PVA/PSSA@ CNTs-SC) (Fig. 9). Due to the insertion of the carboxylic group, which induces the tying and alignment of polymer chains, the PVA/PSSA@CNTs-SA-based membranes generally exhibited a more homogeneous surface compared to the non-modified PVA. The microstructure of the prepared membranes was clearly visible in cross-sectional SEM micrographs that display a wax-like appearance in PVA (Fig. 7a, cross-section), while this property transformed to a spongy-like structure in functionalized PVA-based membranes, particularly in the PVA/PSSA@CNTs-SA membrane. The process of functionalization and cross-linking that significantly impacts the membrane’s structure and characteristics is illustrated by the noted alterations in both the surface and cross-sectional view^55^. Nevertheless, all modified PVA-based membranes exhibited denser microstructures than the original PVA-based membrane. These results may have arisen from the hydrogen bonds formed between hydrophilic groups in the polymer chains and the cross-linking reaction involving polymer chains and functional materials^77^. Conversely, Figs. 8 and 9 illustrate the morphological topographies of the PVA/PSSA@CNTs-CA and PVA/PSSA@CNTs-SC membranes. These micrograph features demonstrate that the membranes possess a compact and homogeneous structure with a rougher surface, unlike the pristine PVA membrane, which displays a smooth, crack-free surface and exhibits no phase separation.

These characteristics may be associated with strong interfacial interactions and compatibility between PVA/PSSA@CNTs and SA or CA. This advantageous property can protect PVA structures from significant damage that could undermine their reinforcement role in the membrane. As a result, this new feature enhances the protonic conductivity of the membranes by forming microchannels for water and hydrogen ions in the PVA/PSSA@CNTs-CA, PVA/PSSA@CNTs-SA, and PVA/PSSA@CNTs-SC composite membranes^78^. It is important to note that surface pores also appeared on the micrograph’s surface but did not extend across the membrane; this could explain why protons pass through the membrane without resistance and methanol crossover is avoided.

### Thermal stability and mechanical properties of the membranes

#### Thermal stability

**Fig. 10:**
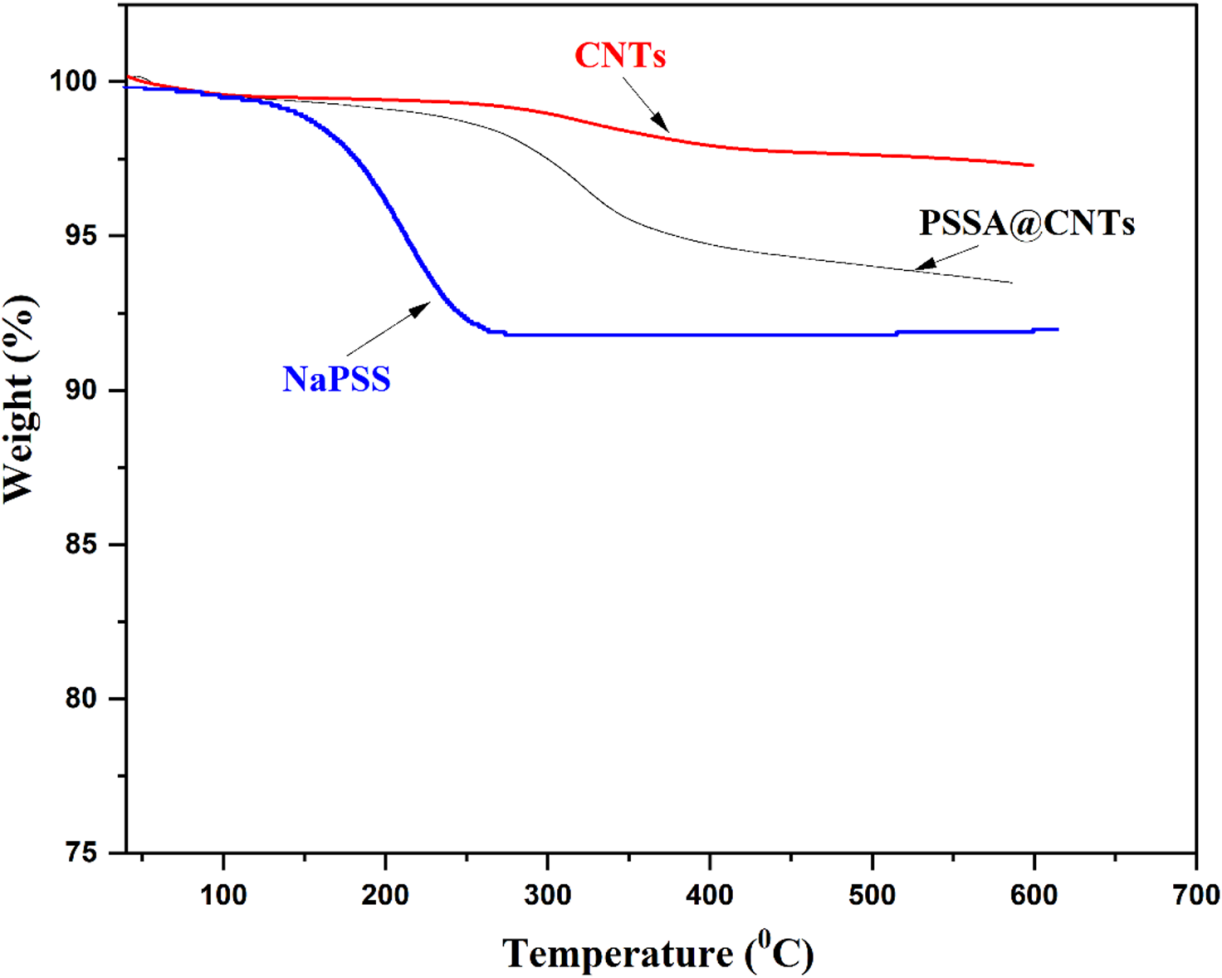
TGA of CNTs, PSSA and PSSA@CNTs.

**Fig. 11:**
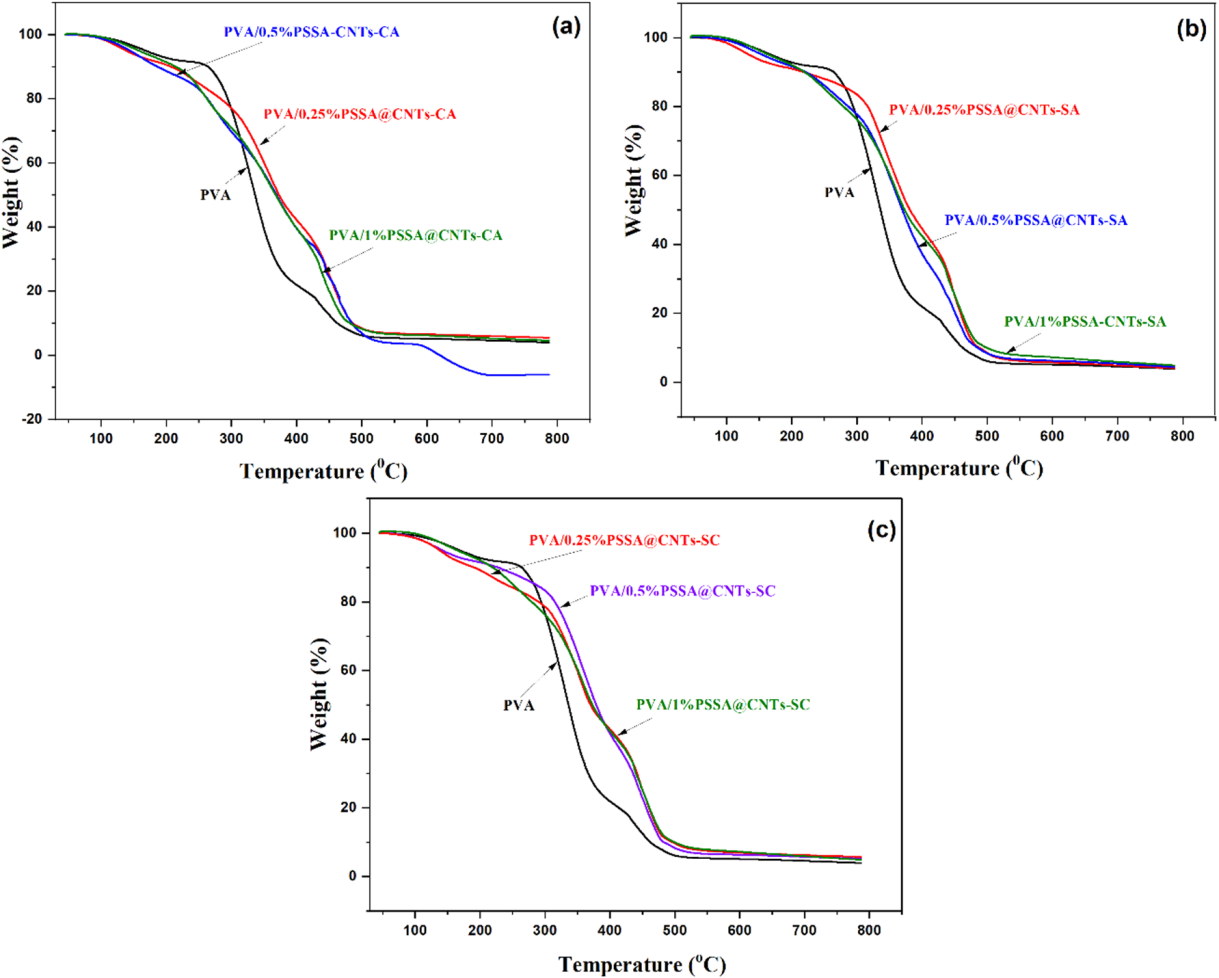
TGA of pristine PVA and cross-linked PVA/PSSA@CNTs membranes.

Thermal stability is a crucial characteristic of proton exchange membranes (PEM). Incorporating PSSA@CNTs into the polymer matrix usually improves thermal stability. The TGA patterns of CNTs, NaPSS, and PSSA@CNTs are presented in Fig. 10. Up to 600 °C, CNTs demonstrate thermal stability. Additionally, the NaPSS polymer exhibits three degradation losses. The initial loss, which can occur at temperatures up to 170 °C, is due to sulfonic acid groups’ breakdown and heat dehydration. The decomposition loss starting at 450 °C may result from the degradation of the primary polymer chain. In contrast, the second loss between 400 and 450 °C is likely attributed to the breakdown of sulfonic acid groups. Conversely, PSSA@CNTs show a significant weight loss at 150 °C, indicating the onset of PSSA degradation. This observation confirms that PSSA is present on the surface of CNTs^79,80^. Three primary stages of thermal degradation can be observed in the PVA thermograms. The first stage, linked to the loss of adsorbed water molecules, occurs between 70 and 200 °C. In contrast, the second stage, occurring between 230 and 340 °C, reflects the thermal degradation of PVA chains with broken -OH groups, as indicated by a peak. The third stage, marked by a residual mass of 1.05%, occurs between 340 and 450 °C and is associated with the carbonization and degradation of the polymer^81,82^. The TGA of the prepared crosslinked PVA/PSSA@CNTs membranes is expected to provide superior thermal and tensile stability compared to the PVA membrane due to the incorporation of PSSA@CNTs and effective crosslinking between PVA/PSSA@CNTs and CA or SA crosslinkers, as illustrated in Fig. 11. All the membranes show three distinct phases of weight loss. The initial weight loss, due to the evaporation of absorbed water, occurs below 160 °C. The second weight loss, resulting from the detachment of sulfonic acid groups from the PSSA@CNTs, occurs within a specific temperature range of 275–395 °C. The third weight loss occurs at around 490 °C, resulting from the disintegration of the polymer backbone^83–85^. Compared to the pristine PVA membrane, the composite PVA/PSSA@CNTs membranes exhibited more significant weight loss during the initial phase, suggesting enhanced water absorption. This finding aligns with the subsequent water absorption test results due to the improved water absorption capability of PSSA@CNTs. Interestingly, when the PVA/PSSA@CNTs-SA composite membranes were heated to 600 °C, they lost more mass than the PVA membrane. This occurred because the -OH, C = O, and -COOH groups at the ends of the SA and CNTs broke down, and water molecules were absorbed into the membranes. Overall, the TGA results indicate that all membranes are thermally stable at temperatures up to 290 °C. This demonstrates that they can function as PEMs operating at a target temperature of less than 120 °C.

#### Mechanical properties

**Fig. 12:**
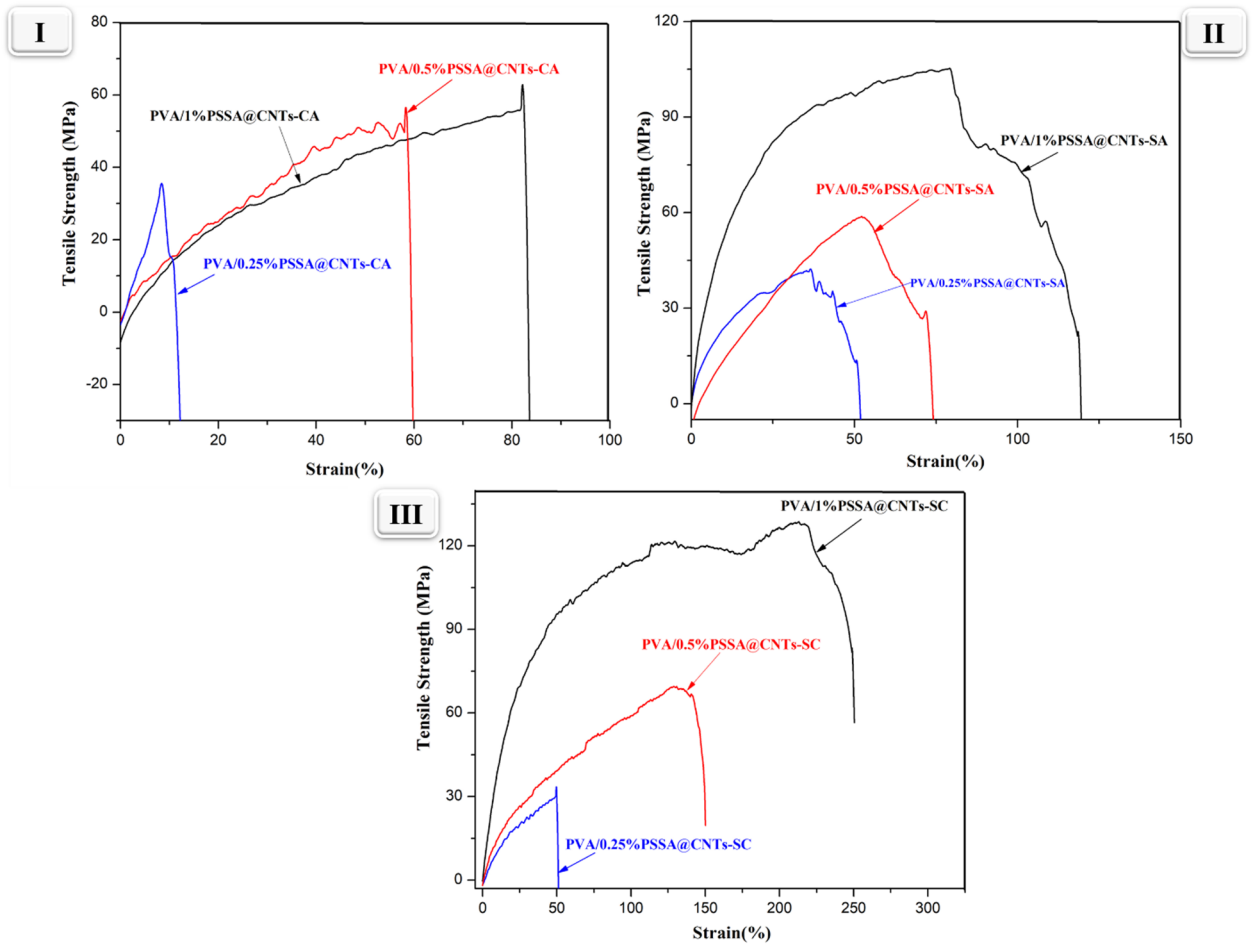
(**I**) Stress strain curve of PVA/PSSA@CNTs-CA, (**II**) Stress strain curve of PVA/PSSA@CNTs-SA, (**III**) Stress strain curve of PVA/PSSA@CNTs-SC.

One of the most important considerations when assessing the potential sustainability of PEM is the mechanical properties. Tensile strength (TS) and Strain (%) of cross-linked PVA/PSSA@CNTs membranes were tested, and the findings are reported in Table 1; Fig. 12. Compared with pristine PVA polymer, increasing the molar ratio of PSSA@CNTs in PVA/PSSA@CNTs membranes causes increased tensile stress due to the reinforcing effect of CNT with a high aspect ratio. Further TS values were modified by incorporating SA, CA, and SC as chemical crosslinkers^86,87^. In the case of PVA/PSSA@CNTs-CA polyelectrolyte membranes, the tensile properties gradually increased with increasing the quantity of the PSSA@CNTs. Hence, the TS of the PVA/0.25%PSSA@CNTs-CA, PVA/0.5%PSSA@CNTs-CA, and PVA/1%PSSA@CNTs-CA membranes were 34.95, 55.29, and 62.75, respectively. Moreover, the tensile characteristics increased when SA was used as a crosslinker, increasing from 43.10 MPa for PVA/0.25%PSSA@CNTs-SA to 68.67 and 104.87 MPa for PVA/0.5% PSSA@CNTs-CA and PVA/1% PSSA@CNTs-CA, respectively. This impact was probably attributable to the physical interaction between PSSA@CNTs and the polymer chains, which resulted in increased entanglements at 1 wt%. Moreover, Strain% was upgraded from 36.65 to 79.31 by integrating PSSA@CNTs into the PVA/PSSA@CNTs, corresponding to an increase of about 50%. This indicated that the association between the PSSA@CNTs and the PVA polymer matrix was robust; hence, the composite membranes demonstrated superior strength compared to the unmodified membrane. In this approach, the enhancement of mechanical performance by adding PSSA@CNTs into the prepared membranes^88,89^. Furthermore, the Tensile strength and Strain% of the PVA/PSSA@CNTs-SC membranes increase from 37.06 to 126.79 MPa wt% because mixing of CA and SA enhances the ionic cross-linking between membrane components as shown in Figs. 2 and 3 which presents the chemical interaction between PVA and PSSA@CNTs to form an ester linkage (RCOOR) and increasing the quantity of PSSA@CNTs causing an increase in the amount of –SO_3_H groups, more polymeric segmental restricting, and high adhesion between polymeric matrix and chemical cross-linkers. Therefore, the prepared cross-linked polymeric membranes can be used as PEMs.

**Table 1:** Tensile strength and Strain (%) of cross-linked PVA/PSSA@CNTs -based membranes.

Membrane Code	Tensile Strength (MPa)	Strain (%)
PVA	15.14	6.87
PVA/0.25% PSSA@CNTs-CA	34.95	8.13
PVA/0.5% PSSA@CNTs-CA	55.29	58.62
PVA/1% PSSA@CNTs-CA	62.75	82.08
PVA/0.25% PSSA@CNTs-SA	43.10	36.65
PVA/0.5% PSSA@CNTs-SA	68.67	52.42
PVA/1% PSSA@CNTs-SA	104.87	79.31
PVA/0.25% PSSA@CNTs-SC	37.06	49.98
PVA/0.5% PSSA@CNTs-SC	69.84	129.46
PVA/1% PSSA@CNTs-SC	126.79	216.36

### Structural properties of the prepared membranes

#### FT-IR spectral analysis

**Fig. 13:**
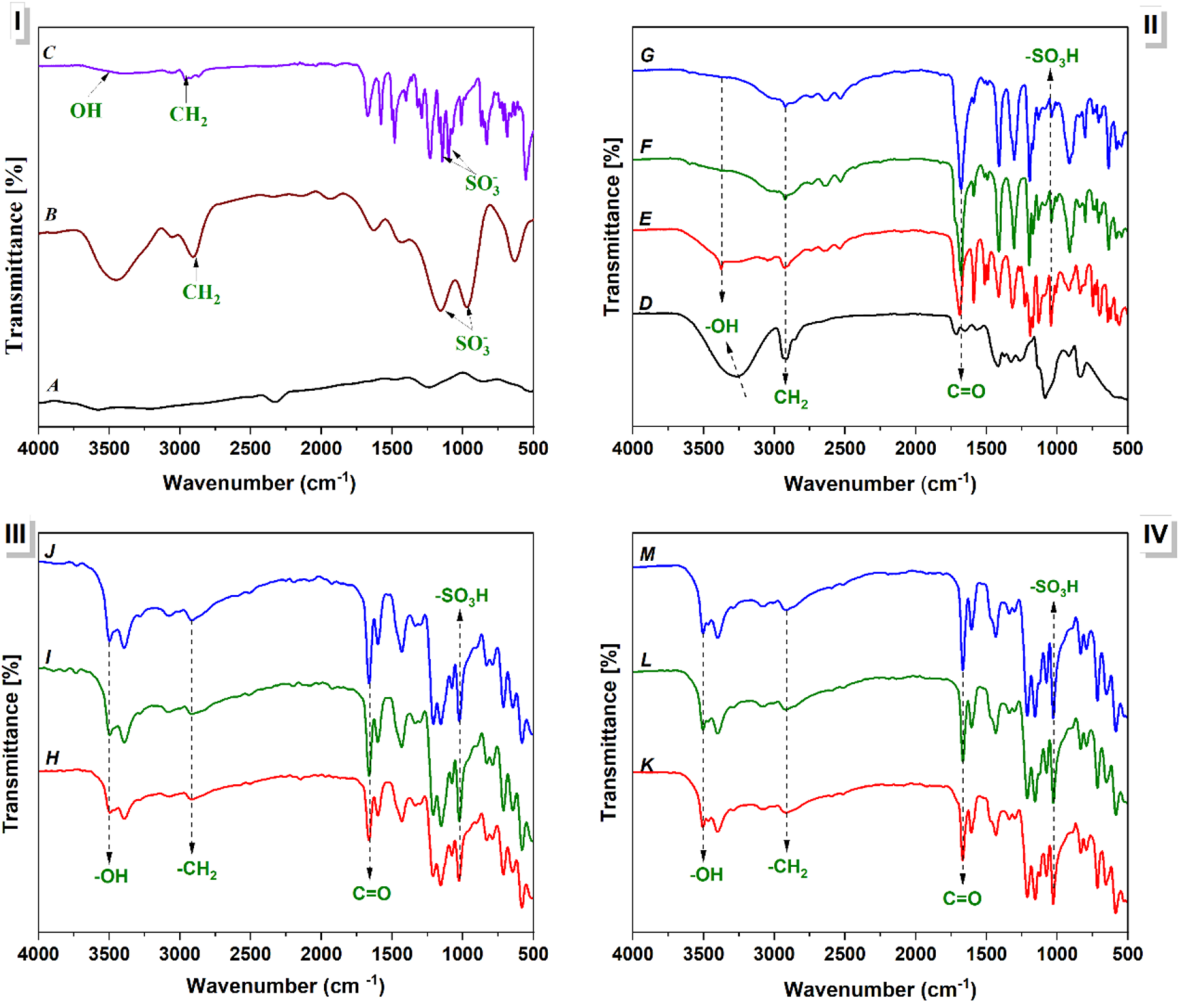
(**I**) FT-IR spectra of (A) CNTs, (B) NaPSS, and (C) PSSA@CNTs, (**II**) FT-IR spectra of (D) Pristine PVA, (E) PVA/0.25%PSSA@CNTs-CA, (F) PVA/0.5% PSSA@CNTs-CA, and (G) PVA/1%PSSA@CNTs-CA (**III**) FT-IR spectra of (H) PVA/0.25%PSSA@CNTs-SA, (I) PVA/0.5%PSSA@CNTs-SA, and (J) PVA/1%PSSA@CNTs-SA (**IV**) FT-IR spectra of (**K**) PVA/0.25%PSSA@CNTs-SC, (L) PVA/0.5%PSSA@CNTs-SC, and (M) PVA/1%PSSA@CNTs-SC-based Polyelectrolyte membranes.

Figure 13 displays the FT-IR spectrum analysis of the synthetic cross-linked PVA/PSSA@CNTs membranes. The investigation reveals the chemical interactions between PVA, PSSA@CNTs, and several varieties of chemical cross-linkers, including CA, SA, and a combination of both (SC). The FT-IR spectra of CNTs (A), NaPSS (B), and PSSA@CNTs (C) are shown in Fig. 13I. The C = C stretching of the graphitic structure is responsible for the distinctive peaks in the CNT spectra that appear at about 1600 cm⁻¹. The presence of sulfonated polystyrene is indicated by the notable peaks in the NaPSS spectra at approximately 1180 cm⁻¹ and 1030 cm⁻¹, which correspond to the symmetric and asymmetric stretching vibrations of the sulfonate (-SO₃⁻) groups. Following functionalization, the PSSA@CNTs spectrum (C) displays increased sulfonate peaks and a sizable -OH stretching band of about 3300 cm⁻¹, indicating that PSSA was successfully coated onto the CNTs. Figure 13II displays the FT-IR spectra of pristine PVA (D) and PVA/PSSA@CNTs membranes with varying PSSA@CNT concentrations using citric acid as a chemical crosslinker (E, F, G). The virgin PVA spectrum (D) shows characteristic -OH stretching at around 3300 cm⁻¹, -CH₂ stretching at about 2940 cm⁻¹, and a recognizable C = O stretching band at about 1730 cm⁻¹^90,91^. After integration and cross-linking with CA, the spectra demonstrate a progressive increase in the intensity of the (-SO₃H) peaks (~ 1180 cm⁻¹ and ~ 1030 cm⁻¹) with increasing PSSA@CNTs loading. The increased C = O stretching band at roughly 1730 cm⁻¹, which denotes the formation of ester bonds, confirms the successful cross-linking between the carboxyl (-COOH) groups of CA and/or hydroxyl (-OH) groups of PVA or functionalized CNTs reactions^92^. This structural alteration is expected to enhance the membrane’s ionic conductivity and mechanical stability.

The FT-IR spectrum analysis of membranes based on PVA/PSSA@CNTs crosslinked with s (SA) at varying molar concentrations of PSSA@CNTs (H, I, J) is shown in Fig. 13III. These spectra show necessary functional group vibrations, such as -OH stretching (~ 3300 cm⁻¹), -CH₂ stretching (~ 2940 cm⁻¹), and C = O stretching (~ 1730 cm⁻¹), which are similar to the CA-crosslinked membranes. Significantly, enhanced sulfonation is shown by the strength of the sulfonate (-SO₃H) peaks at ~ 1180 cm⁻¹ and ~ 1030 cm⁻¹, increasing with more significant PSSA@CNTs loading. SA-based membranes have significantly less noticeable C = O peaks than CA-based membranes, indicating variations in esterification efficiency brought on by the crosslinker’s chemical composition. The retention of proton-conducting sites, which are essential for DMFC performance, is confirmed by the existence of strong -SO₃H bands. The FT-IR spectrum analysis of PVA/PSSA@CNTs-based membranes crosslinked with citric acid and succinic acid (SC) combination at various PSSA@CNTs molar concentrations (K, L, M) is shown in Fig. 13IV. The spectra maintain the distinctive functional groups observed in earlier membranes, including the bands for -OH (~ 3300 cm⁻¹), -CH₂ (~ 2940 cm⁻¹), C = O (~ 1730 cm⁻¹), and -SO₃H (~ 1180 cm⁻¹ and ~ 1030 cm⁻¹)^93^.The distinct -SO₃H and C = O peaks show that the hybrid crosslinking procedure seems to improve both the sulfonation and esterification processes. This implies better compatibility between the conductive filler and the polymer matrix, which could increase methanol resistance, proton conductivity, and membrane stability^94^. Subsequently, the FT-IR analysis demonstrates that PSSA@CNTs were successfully integrated into the PVA polymer network, with specific chemical interactions influenced by the crosslinker preference. Higher PSSA@CNT concentration indicates increased proton conductivity, crucial for DMFC performance, as evidenced by the enhancing sulfonic acid (-SO₃H) vibrations. The differences in crosslinking efficiency between CA, SA, and SC impact membrane flexibility, stability, and ion transport. These results lend credence to the potential of polyelectrolyte membranes as viable options for DMFC applications.

#### Raman spectroscopy

**Fig. 14:**
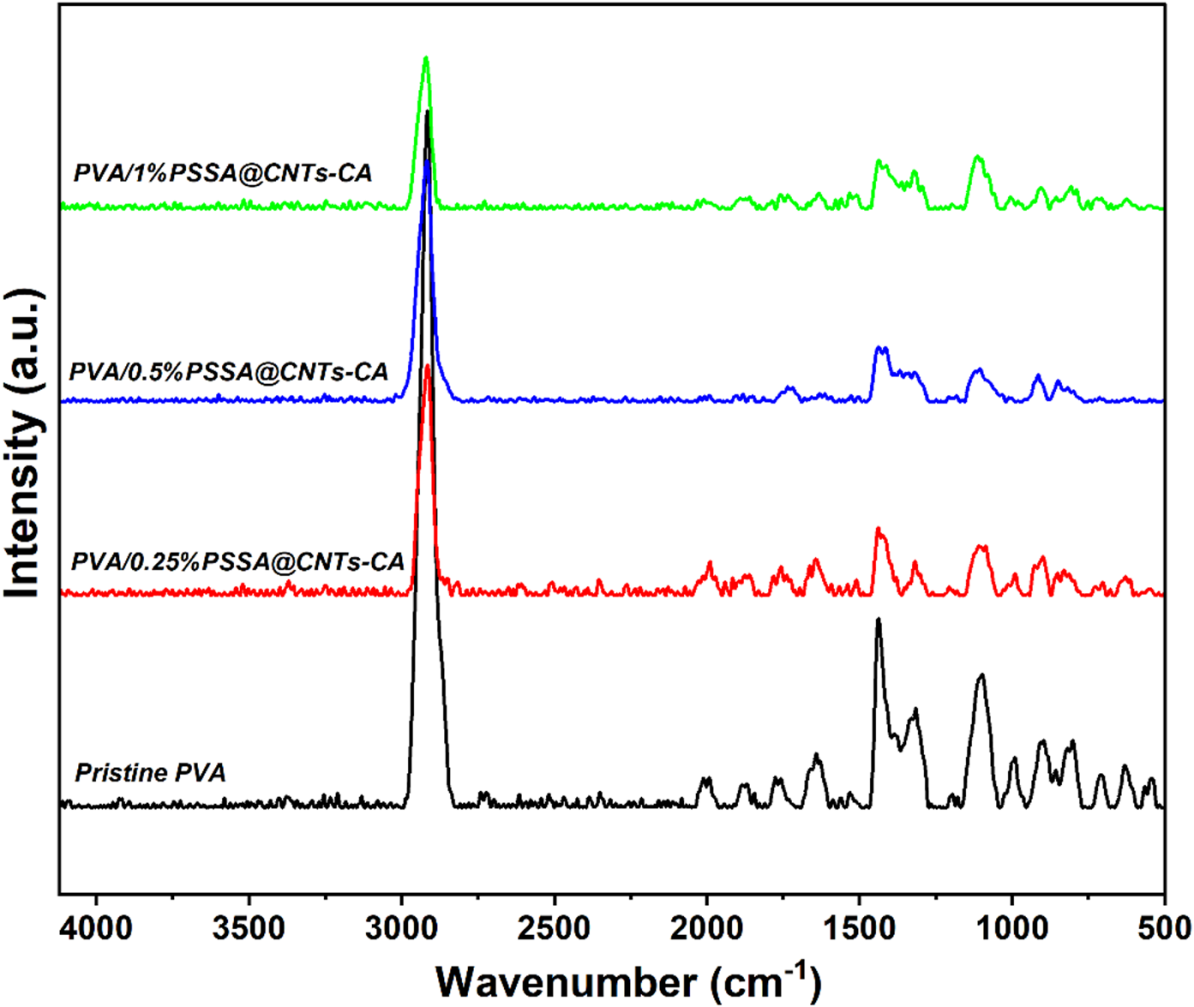
Raman spectra of the prepared PEMs based on (PVA/PSSA@CNTs-CA) with different molar concentrations of PSSA@CNTs.

**Fig. 15:**
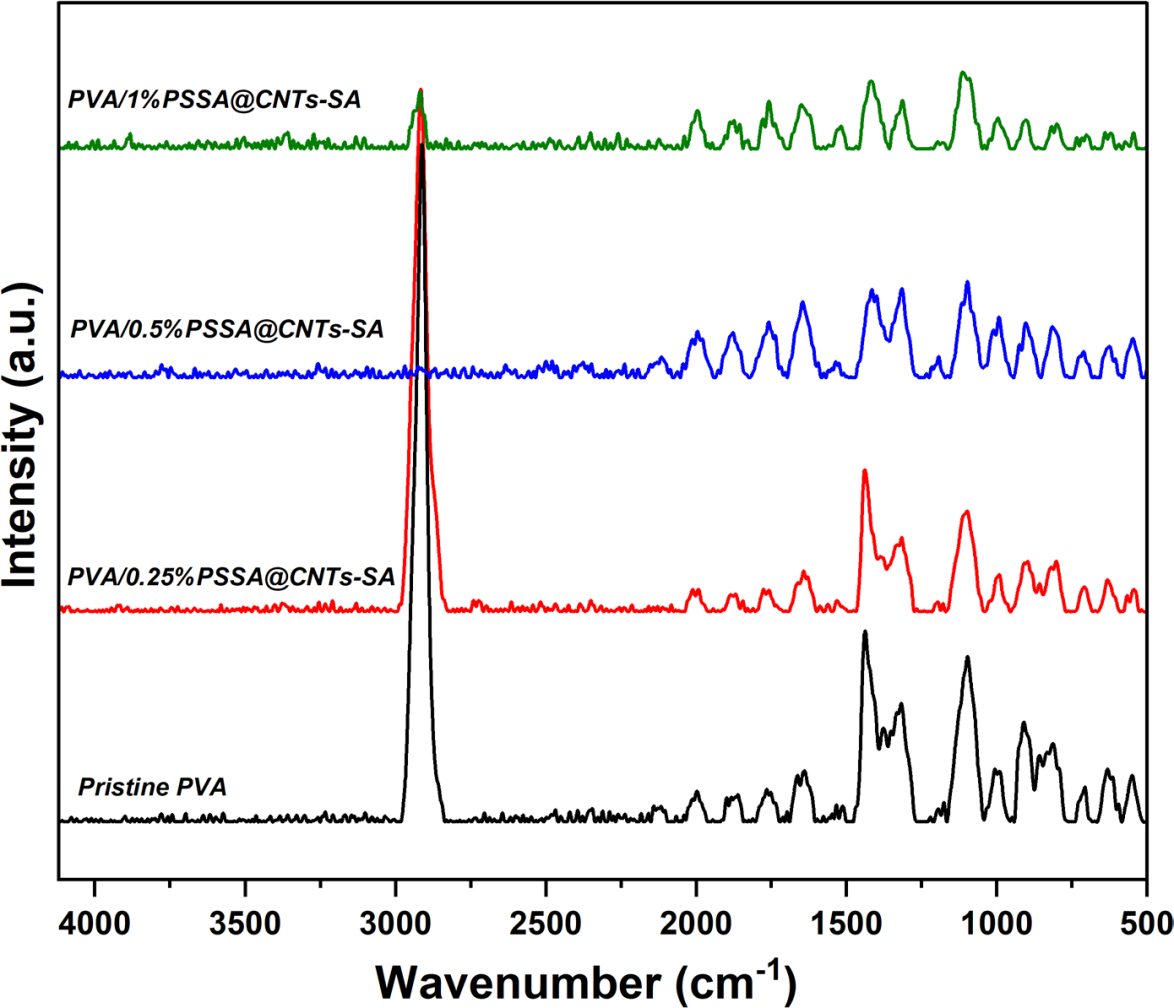
Raman spectra of the prepared PEMs based on (PVA/PSSA@CNTs-SA) with different molar concentrations of PSSA@CNTs.

**Fig. 16:**
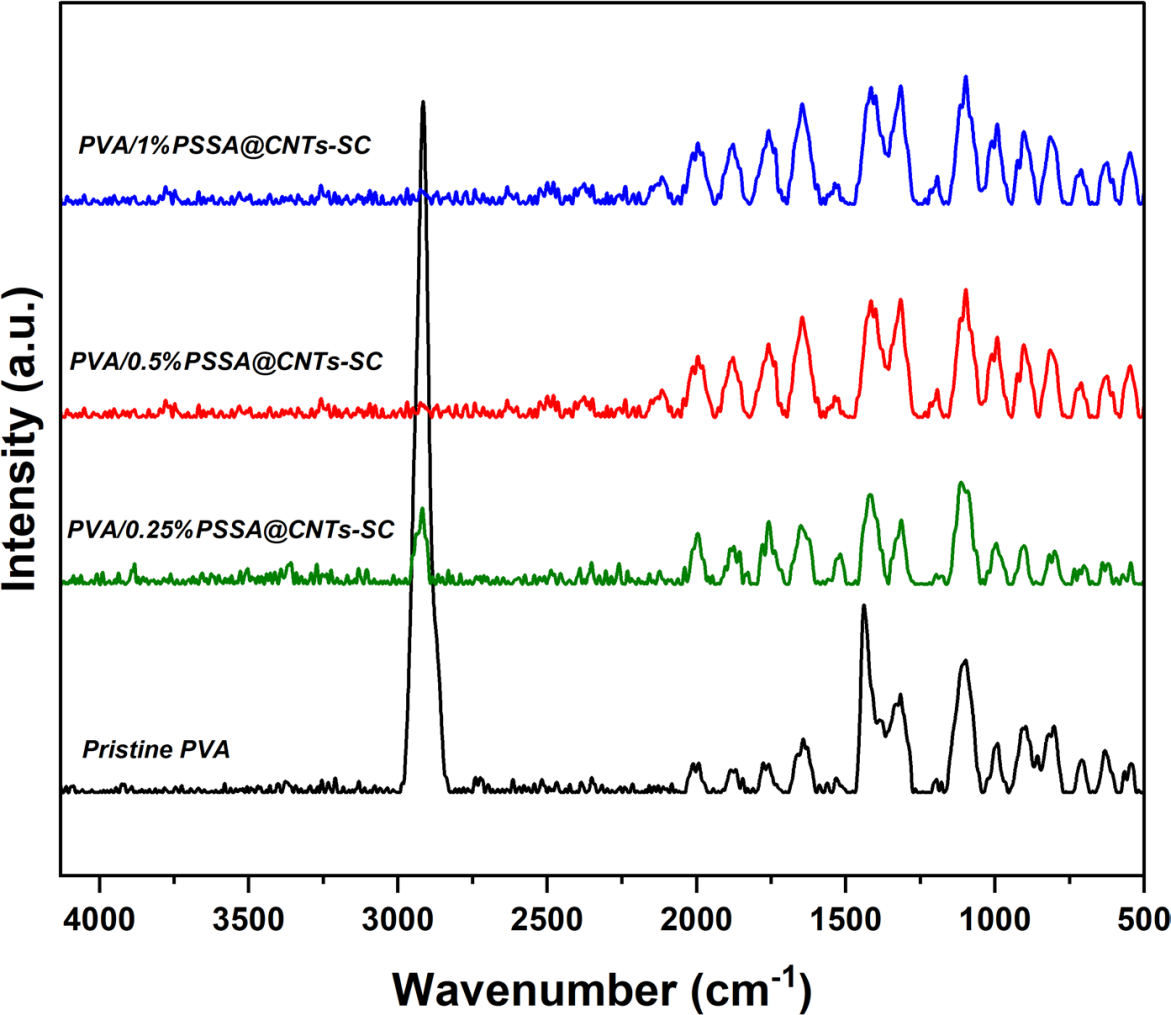
Raman spectra of the prepared PEMs based on (PVA/PSSA@CNTs-SC) with different molar concentrations of PSSA@CNTs.

Figure 14 shows how the nondestructive Raman spectroscopy technique was used to uncover further information about the membrane microstructure. The Raman spectra of the PVA polymer membrane showed several strong characteristic scattering peaks. The strong peak at 1440 cm^− 1^ was due to the O–H bending and C–H bending. Also, the further vibrational peaks that appear at 854 and 917 cm^− 1^ were due to the stretching of the C–C bond. Furthermore, there were some scattering weak peaks due to the C–C stretching and C–O stretching that presented at 1060, 1090, and 1145 cm^− 1^^95,96^. Raman spectra of (PVA/PSSA@CNTs-SA) polyelectrolytic membranes are shown in Fig. 15. The absorbed peaks at 1106 cm^− 1^ and 1445 cm^− 1^ appear in all Raman curves due to the vibration of C–C and C-H bonds, respectively. The C-C in the PVA polymeric chain was attributed to the other strong vibrational peak, which showed about 900 cm^− 1^. The asymmetric vibration of C-O-C bonds at 1099 cm^− 1^ has produced additional signals that support the chemical cross-linking processes. Furthermore, a strong valance C = O group band appears at 1634 cm^− 1^, and its intensity rises as PVA is functionalized with SA, CA, and/or SC, as shown in Fig. 16.

### Elemental analysis of the prepared membranes

The composition of prepared membranes is characterized using elemental analysis to verify the incorporation of PSSA@CNTs and PVA membranes, and the acquired results have been listed in Table 2. Notably CHNS analysis of pristine PVA reveals the presence of C (47.62%), H (7.45%), and O (44.55%), confirming that it exhibits high purity and uniform distribution. The membranes’ oxygen content was theoretically calculated. In the case of pristine PVA, for instance, (C, H, and O) accounts for all of the PVA content. Consequently, if the instrument’s experimental C&H content is calculated to be 55%, the oxygen percentage will be 45%^97,98^. As the molar proportion of PSSA@CNTs in the membrane increases, the sulfur concentration increases, indicating that the amount of –SO_3_H groups linked to the PSSA@CNTs backbone has grown. CHNS results of cross-linked PVA/PSSA@CNTs films prove that they possess all the elements that make up the structure of polymer (C, H &S). Results reveal that increasing PSSA@CNTs concentration, Sulfur, and oxygen concentration increasing from and carbon quantity is deceased for each group by introducing of the chemical cross-linker into the PVA/PSSA@CNTs that leads to ether bonding or esterification through the removal of H2O molecules, causing a decrease in hydrogen ratios. This is evident that occurring of esterification reaction as mentioned in FT-IR spectroscopy^99,100^. As a result, the modest shift in C/H ratios for each group suggests membrane composition stability after adding a chemical cross-linker.

**Table 2:** Elemental analysis of the prepared PVA/ PSSA@CNTs-based polyelectrolytic membranes.

Membrane	O% (Theoretical)	C%	H%	S%	C/O % (Theoretical)	C/H% (Theoretical)
Pristine PVA	44.93	47.62	7.45	-	1.05	6.39
PVA/ 0.25% PSSA@CNTs-CA	48.12	43.10	7.57	1.28	0.89	5.82
PVA/ 0.5% PSSA@CNTs-CA	49.42	41.18	7.22	2.18	0.83	5.84
PVA/ 1% PSSA@CNTs-CA	50.22	39.14	7.24	3.10	0.77	5.68
PVA/ 0.25% PSSA@CNTs-SA	45.43	46.31	6.46	1.53	0.99	6.65
PVA/ 0.5% PSSA@CNTs-SA	47.26	44.19	6.00	2.54	0.94	7.33
PVA/ 1% PSSA@CNTs-SA	47.75	42.09	5.64	4.52	0.88	7.90
PVA/ 0.25% PSSA@CNTs-SC	44.31	46.39	7.02	2.06	1.04	6.60
PVA/0.5% PSSA@CNTs-SC	45.85	46.07	6.08	2.07	1.00	7.57
PVA/ 1% PSSA@CNTs-SC	47.15	43.49	6.21	3.15	0.92	7.00

### Water and methanol uptake, dimensional stability, gel fraction of the membrane

Water uptake (WU) and methanol uptake (MU) are significant parameters overseeing proton transportation behavior and dimensional stability in polyelectrolyte membranes whereas satisfactory hydration facilitates proton conduction, while excessive swelling may impair mechanical integrity and long-term durability. As summarized in Table 3, pristine PVA membranes transformed into a gel upon soaking, indicating insufficient structural stability in aqueous media. In contrast, all cross-linked PVA/PSSA@CNT membranes maintained structural integrity, confirming the effectiveness of the crosslinking. The results showed that the WU of the cross-linked membranes, increased gradually with increasing PSSA@CNT molar ratio, which can be attributed to the higher concentration of hydrophilic sulfonic acid (–SO₃H) groups introduced by PSSA@CNTs. The WU values ranged from 38.86 ± 5% (PVA/0.25% PSSA@CNTs-SA) to 67.25 ± 5% (PVA/1% PSSA@CNTs-SC). In addition to relatively moderate water absorption, the crosslinked membranes showed dimensional stability where in-plane swelling (ΔA, %) remained limited to approximately 11.33 ± 0.3% for PVA/ 0.25% PSSA@CNTs-SA and 13.75 ± 0.4% for PVA/1% PSSA@CNTs-CA, which is much lower than the swelling of Nafion-117 (35% in-plane swelling), and Through-plane swelling (ΔT, %) was limited to 14.45 ± 0.3 for PVA/ 0.25% PSSA@CNTs-SA, and 18.01 ± 0.1 for PVA/1% PSSA@CNTs-CA, which is very close to the swelling of Nafion-117 (15% through-plane swelling). These results indicate that the cross-linked network effectively accommodates water within the membrane without excessive dimensional expansion. It is worth noting that the crosslinking interaction between the hydroxyl groups in polyvinyl alcohol (PVA) and the multifunctional carboxylic acid groups in the crosslinking agents forms a three-dimensional covalent network that restricts the movement of the polymer chains and reduces deformation caused by water absorption. Furthermore, PSSA@CNT incorporation provides additional physical reinforcement through polymer–filler interfacial interactions, reducing free volume and stabilizing membrane dimensions^44,101^. Moreover, the values for methanol uptake ranged from 9.37 ± 2% for the PVA/1% PSSA@CNTs-SA to 12.14 ± 3% for the PVA/0.25% PSSA@CNTs-CA. These values were much lower than the methanol uptake of the Nafion-117 (22%), which means that the developed polyelectrolytic membranes have better resistance to methanol crossover. The decrease is due to the cross-linked structure becoming denser and the diffusion channels in the composite membranes becoming limited. The gel fraction (%) of the developed PEMs, an indicator of crosslinking density, was evaluated as shown in Table 3 and decreased slightly with increasing PSSA@CNTs loading, indicating a minor reduction in effective crosslinking density. The results exhibit that, the highest gel fraction was recorded for PVA/0.25% PSSA@CNTs-CA (81.16 ± 2.55%), while the lowest was recorded for PVA/1% PSSA@CNTs-SA (60.63 ± 2.20%), reflecting the effect of different chemical crosslinkers on network formation. This behavior may be attributed to physical obstruction and partial engagement of PSSA@CNTs functional groups with the cross-linkers, which may limit the formation of a fully crosslinked network, consistent with the observed increase in water uptake and dimensional Stability^102,103^. Additionally, based on the preparation method (1.5 g PVA in 25 mL solution, 6 wt%, and 0.15 g crosslinker, 10 wt% relative to PVA content), the molar ratio of carboxylic (–COOH) to hydroxyl (–OH) groups was calculated as a stoichiometric estimation of the theoretical cross-linking potential for each system. Using the PVA repeating unit (44 g mol⁻¹ per –OH), the COOH/OH ratios were 0.069 for CA, 0.075 for SA, and 0.072 for the SC system. These values indicate a moderate and controlled cross-linking density, consistent with the observed balance between mechanical reinforcement and proton conductivity^104^. Collectively, the combined effects of controlled chemical cross-linking and the addition of conductive nanomaterials create a good balance between water absorption, dimensional stability, and resistance to methanol. These results make those membranes good candidates for stable operation of a proton exchange membrane (PEM)^105,106^.

### Water contact angle (WCA)

**Fig. 17:**
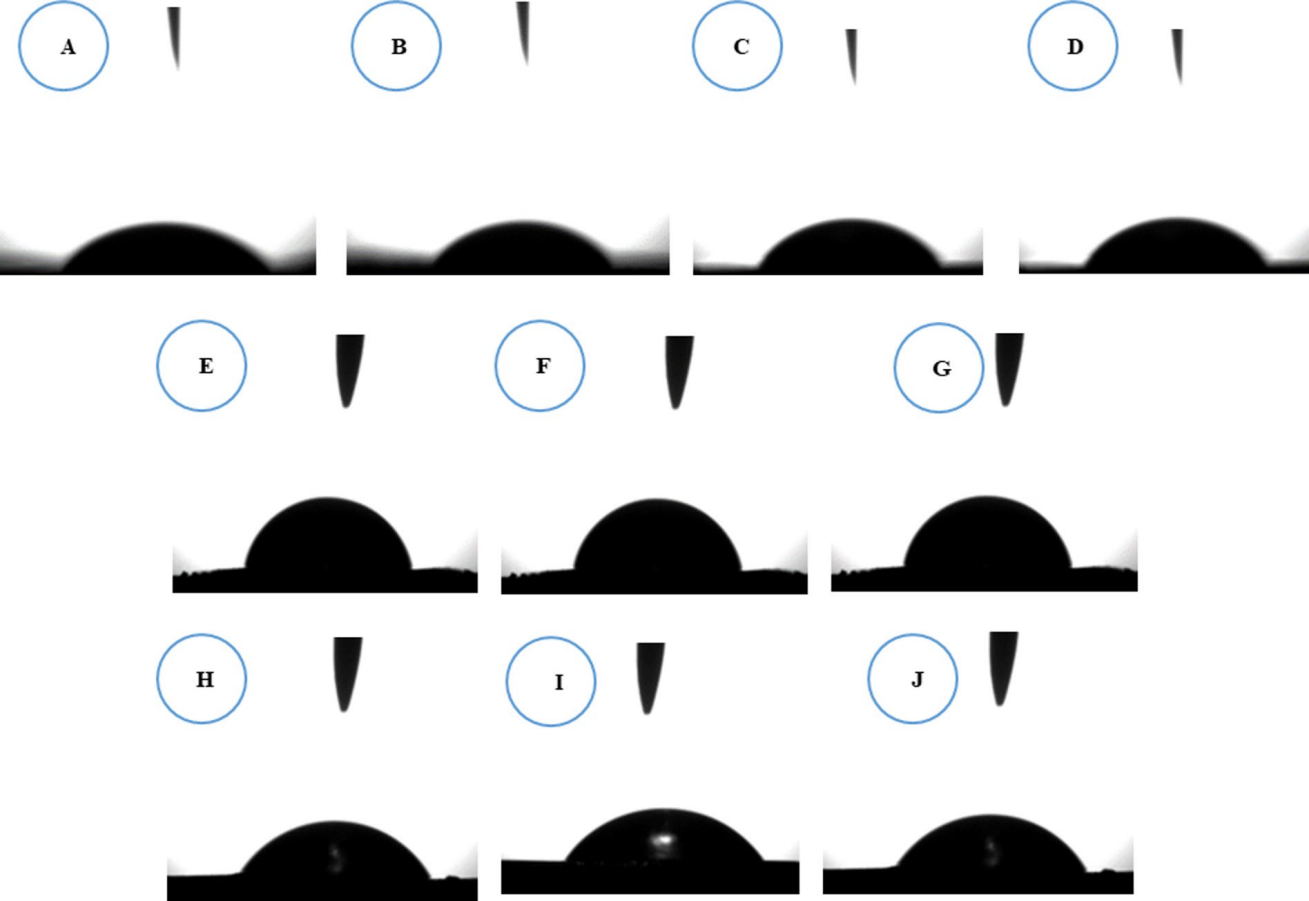
The digital photographs of water contact angle of (**A**) pristine PVA, (**B**) PVA/0.25%PSSA@CNTs-CA, (**C**) PVA/0.5% PSSA@CNTs-CA, (**D**) PVA/1%PSSA@CNTs-CA, (**E**) PVA/0.25%PSSA@CNTs-SA, (**F**) PVA/0.5%PSSA@CNTs-SA, (**G**) PVA/1%PSSA@CNTs-SA, (**H**) PVA/0.25%PSSA@CNTs-SC, (**I**) PVA/0.5%PSSA@CNTs-SC, and (**J**) PVA/1%PSSA@CNTs-SC-based Polyelectrolyte membranes.

The hydrophilicity of a polyelectrolyte membrane represents its ability to absorb or swell with water and is closely related to water durability, ion exchange capacity (IEC), and proton conductivity. Specifically, this property was evaluated using water contact angle (WCA) measurements. From the data in Fig. 17, the pristine PVA membrane displayed a low contact angle of 34.05°, indicating a highly hydrophilic surface; this behavior is primarily due to the high concentration of free hydroxyl (–OH) groups along the PVA backbone, which encourage hydrogen bonding with water molecules and facilitate immediate droplet spreading. A different behavior was observed upon modification: In contrast, the addition of PSSA@CNTs and crosslinking agent led to a substantial increase in WCA (58–72°), reflecting a relative decrease in surface hydrophilicity. This is due to ester linkage formation between PVA hydroxyl groups and the carboxylic groups of the crosslinkers, which diminishes the number of free polar –OH groups, while also causing structural rearrangement and packing of the polymer network that restricts accessibility of hydrophilic groups at the surface. Besides the slight hydrophobic character imparted by the presence of CNT domains near the surface, increasing the PSSA@CNTs content from 0.25 wt% to 1 wt% resulted in a gradual decrease in WCA due to increasing the density of sulfonic acid (–SO₃H) groups introduced by PSSA@CNTs, which boost surface polarity, facilitating water spreading. This trend was consistent across all modified membranes: for instance, the WCA dropped from 63.52° (PVA/0.25%PSSA@CNTs-CA) to 58.15° (PVA/1%PSSA@CNTs-CA), from 72.15° (PVA/0.25%PSSA@CNTs-SA) to 63.10° (PVA/1%PSSA@CNTs-SA), and from 69.73° (PVA/0.25%PSSA@CNTs-SC) to 60.15° (PVA/1%PSSA@CNTs-SC) membranes upon increasing the PSSA@CNTs content. It is essential to differentiate between surface wettability and bulk hydration behavior. While crosslinking initially increases the WCA, WU and IEC values increase with higher PSSA@CNTs content. This indicates that hydrophilic functional groups are mainly distributed within the bulk of the membrane rather than being confined to the surface. Thus, crosslinking controls the initial increase in WCA, whereas higher PSSA@CNTs loading enhances overall membrane hydrophilicity and hydration capacity^107^.

### Ion exchange capacity (IEC)

The IEC values of the prepared PVA/PSSA@CNTs membranes are presented in Table 3. The kind and quantity of hydrophilic clusters in the polymer backbone affect the PEMs’ capacity to absorb water and ions with a low swelling ratio^108^. Table 3 shows that increasing IEC values in the prepared membranes is due to increasing PSSA@CNTs content into the membrane matrix to reach 3.03 (meq/gm) in the PVA/ 1% PSSA@CNTs-SA membrane. Additionally, it increased from (2.07 to 2.66) (meq/gm) by using a CA crosslinker due to the attendance of extra ion-exchangeable groups (-SO_3_H groups) existing from the PSSA backbone. The membranes with PVA/1% PSSA@CNTs-SA and PVA/1% PSSA@CNTs-SC had the most significant IEC data (2.86 meq/g and 3.03 meq/g, respectively), as cross-linking successfully introduces negative surface charge beside increases the water stability of the prepared membranes. Different chemical cross-linkers were employed to alter the electrochemical characteristics of membranes, which resulted in variations in the IEC results. This can be explained by the fact that the IEC and ionic conductivity of PVA/PSSA@CNTs membranes increased in tandem with the acid concentration.

**Table 3:** Thickness, WCA, WU, in-plane swelling (ΔA), through-plane swelling (ΔT), gel fraction, MU, and IEC of the pristine PVA and cross-linked PVA/ PSSA@CNTs-CA, PVA/ PSSA@CNTs-SA & PVA/PSSA@CNTs-SC-based polyelectrolyte membranes.

Membrane	Thickness (µm)	WCA (°)	WU (%)	In-plane swelling (ΔA, %)	Through-plane swelling (ΔT, %)	Gel Fraction (%)	MU (%)	IEC (meq/gm)
Pristine PVA	81 ± 1	34.05 ± 2	Gel	Gel	Gel	17.54 ± 0.55	17.54 ± 2	0
PVA/ 0.25% PSSA@CNTs-CA	74 ± 2	63.52 ± 3	57.26 ± 4	12.55 ± 0.2	15.31 ± 0.1	81.16 ± 2.55	12.14 ± 3	2.07 ± 0.1
PVA/ 0.5% PSSA@CNTs-CA	77 ± 2	60.42 ± 2	60.51 ± 5	13.17 ± 0.5	16.70 ± 0.2	74.62 ± 2.76	11.04 ± 2	2.54 ± 0.2
PVA/1% PSSA@CNTs-CA	80 ± 3	58.15 ± 1	65.36 ± 3	13.75 ± 0.4	18.01 ± 0.1	71.07 ± 2.92	10.88 ± 3	2.66 ± 0.2
PVA/0.25% PSSA@CNTs-SA	90 ± 2	72.15 ± 1	38.86 ± 5	11.33 ± 0.3	14.45 ± 0.3	68.45 ± 3.05	11.54 ± 2	2.47 ± 0.4
PVA/0.5% PSSA@CNTs-SA	92 ± 2	70.35 ± 1	40.16 ± 4	11.75 ± 0.4	14.98 ± 0.1	63.20 ± 2.36	10.32 ± 2	2.83 ± 0.4
PVA/1% PSSA@CNTs-SA	95 ± 2	63.10 ± 1	45.11 ± 2	12.87 ± 0.7	15.51 ± 0.2	60.63 ± 2.20	9.37 ± 2	3.03 ± 0.4
PVA/0.25% PSSA@CNTs-SC	82 ± 2	69.73 ± 1	60.51 ± 5	11.94 ± 0.4	15.01 ± 0.2	77.12 ± 3.36	11.32 ± 2	2.13 ± 0.4
PVA/0.5% PSSA@CNTs-SC	85 ± 2	65.13 ± 2	65.31 ± 3	12.20 ± 0.4	15.55 ± 0.3	72.45 ± 2.57	10.52 ± 2	2.53 ± 0.4
PVA/1% PSSA@CNTs-SC	87 ± 2	60.15 ± 1	67.25 ± 5	12.34 ± 0.5	15.89 ± 0	69.14 ± 3.15	10.12 ± 2	2.86 ± 0.4
Nafion-117^59^	85	110	50.44	35	15	-	22	0.91

### Proton Conductivity Measurement

**Fig. 18:**
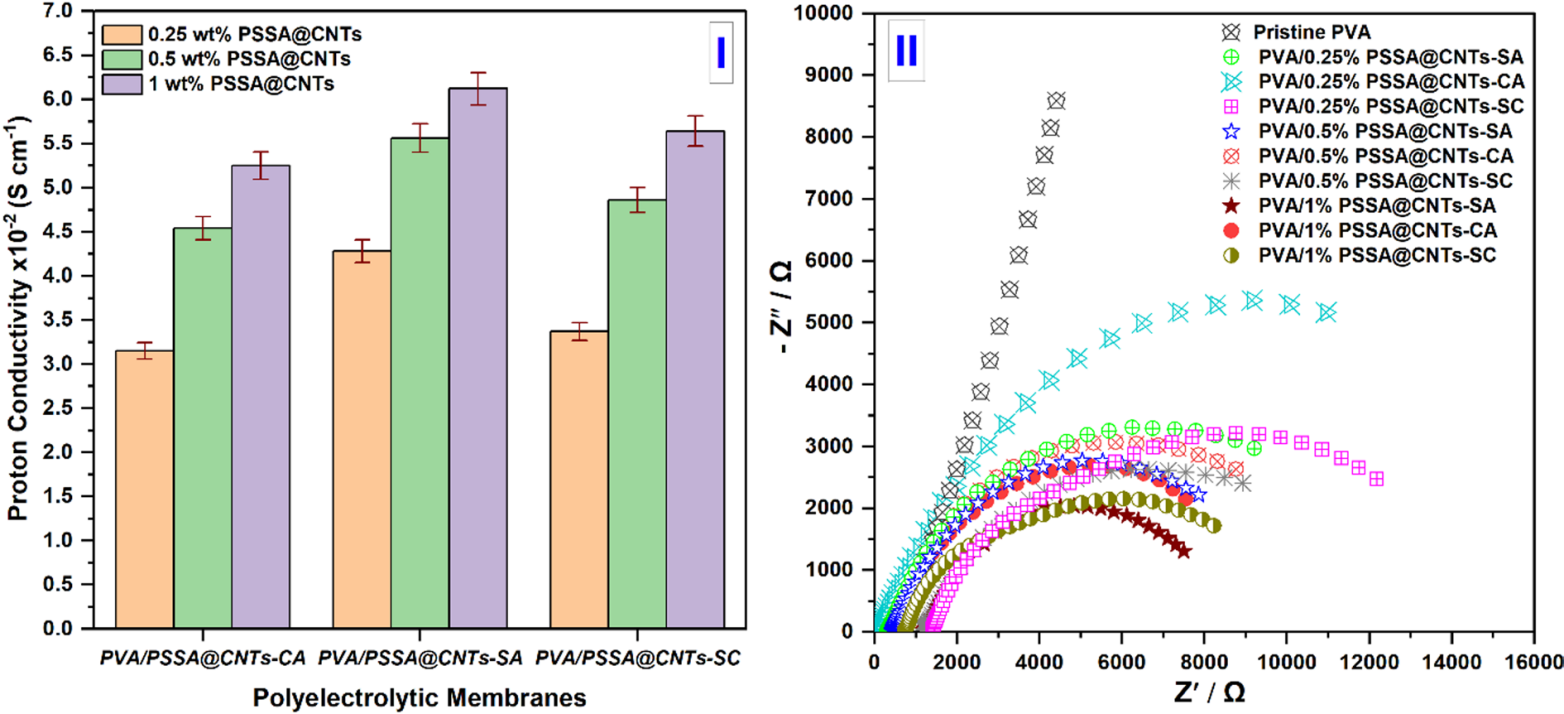
Proton conductivity (**I**) and Simulated Nyquist plots (**II**) of the synthesized cross-linked PVA/PSSA@CNTs membranes with different molar ratios of PSSA@CNTs.

Figure 18I depicts Proton conductivity measurements of synthetic cross-linked PVA/PSSA@CNTs membranes as a function of PSSA@CNTs percentages using different chemical cross-linkers. Due to a lower ratio of transport ions and ionic groups linked to PVA chains that may contain, the unmodified prepared PVA-based membrane exhibits the lowest proton conductivity value equal to (1.34 ± 0.04) x 10^− 4^ S cm^− 1^. Water absorption and IEC data measurements significantly impact membrane ionic conductivity. Increased IEC values facilitate faster proton conduction by reducing the distance between anionic groups, and higher water intake encourages proton transport. By incorporating functionalized carbon nanotubes (PSSA@CNTs) in varying molar concentrations to the polymer matrix, the prepared polyelectrolytic membranes’ proton conductivity was raised compared to pristine membranes. This is because the negative charge of carboxylic groups that can attract protons makes it easier for protons to move through the membrane [94,97]. Furthermore, the proton conductivity increased from (3.15 ± 0.095) x 10^− 2^ S cm^− 1^ to (5.25 ± 0.16) x 10^− 2^ S cm^− 1^ for PVA/PSSA@CNTs-CA, (4.28 ± 0.13) x 10^− 2^ S cm^− 1^ to (6.12 ± 0.18) x 10^− 2^ S cm^− 1^ for PVA/PSSA@CNTs-SA, and (3.37 ± 0.10) x 10^− 2^ S cm^− 1^ to (5.64 ± 0.17) x 10^− 2^ S cm^− 1^ for PVA/PSSA@CNTs-SC when the molar concentration of PSSA@CNTs in PVA/CNTs-based polyelectrolyte membranes increased from 0.25 wt% to 1 wt%. Moreover, the enhancement of proton conductivity may be due to the addition of CA, SA and SC to PVA/PSSA@CNTs-based membranes increases proton conductivity due to the presence of a negative charge that attracts protons and facilitates proton transportation through the membrane; additionally, those conductive materials worked as chemical cross-linkers and provided a proton channel pass way and a large surface area, particularly at functionalized PVA/PSSA@CNTs-SA based membranes with a higher proton conductivity of (6.12 ± 0.18) x 10^− 2^ S cm^− 1^. This followed the same pattern of behavior as the increasing values of IEC. Relationship between the imaginary part of the impedance symbolized (Z″), and the fundamental component of the impedance symbolized (Z′), the Bode and Nyquist plots of synthetic membranes are displayed in Fig. 18II. Studying the polarization resistance to determine the proton conductivity of synthetic membranes requires using the Nyquist plot. The specimens demonstrate how frequency negatively affects the impedance’s pattern of activity. In this instance, all samples showed a circuit of resistance and capacitors connected in parallel. The linear behavior of the Cole-Cole plot is recognized as suggesting resistance and capacitance linked in the series. Samples can be great candidates for use as PEMs because they all have improved and exhibit semicircle-shaped behavior at higher frequencies. By adding different crosslinkers into the membrane matrix, such as SA, CA, and SC, conductivity increased compared to an unmodified membrane. The development of a single arc in the impedance plot indicated the presence of one type of relaxation mechanism.

### Oxidative stability analysis of the prepared PEMs

**Fig. 19:**
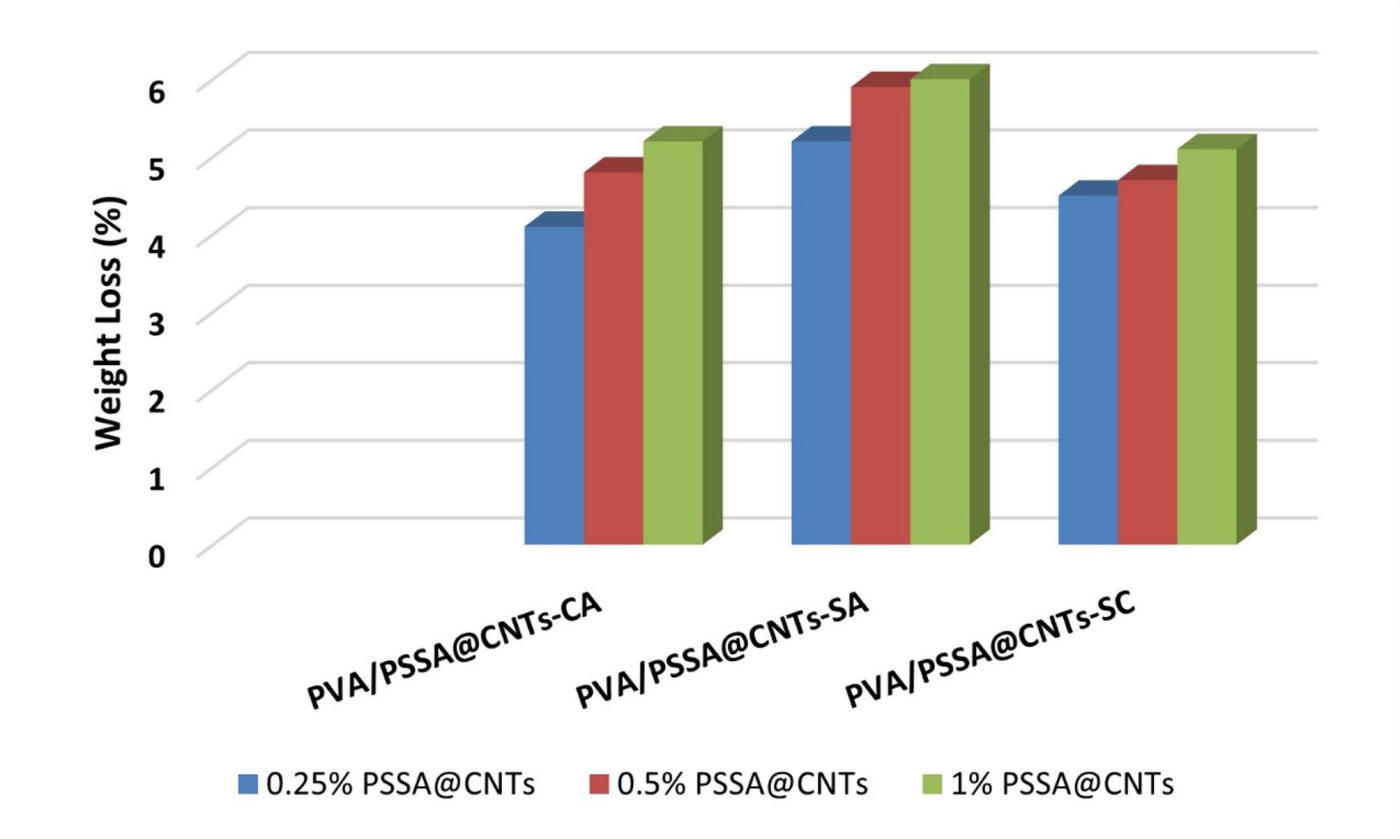
Oxidative stability of cross-linked PVA/PSSA@CNTs membranes.

Overall, the polyelectrolyte membranes predominantly require having a high degree of oxidative stability to be suitable for use in a variety of electrochemical applications, including fuel cells. Additionally, the oxidative stability of the membranes is just as crucial as their thermal and mechanical stability. Figure 19 shows the findings of an investigation into the stability of PVA/PSSA@CNTs membranes in Fenton’s reagent. All prepared polyelectrolytic membranes typically exhibit excellent oxidative stability. Oxygen-containing groups like –COOH and –OH in the crosslinking membranes reduce the hydrophilicity of PEMs. Further, when utilized in fuel cell applications, this shields the hydrophilic sulfonic groups of PSSA@CNTs in the PVA polymer backbone from attacks by OOH and OH radicals on the electrodes. As a result, the produced membranes offer an alternate method of creating ion-exchange membranes in which the resistance of the membrane to oxidation is unaffected by an increasing of ion-exchanging groups quantities. Moreover, the membrane sample held together during the test, indicating the synthetic membrane’s higher level of chemical stability^62^.

### Methanol permeability

**Fig. 20:**
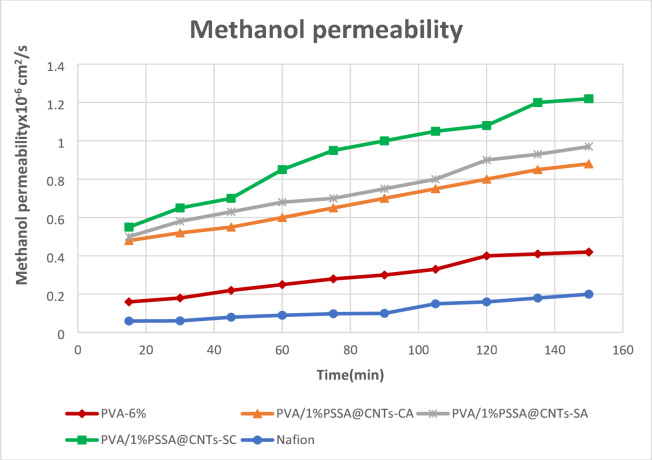
Methanol concentration for PVA/PSSA@CNTs- based poly electrolytic membranes.

Figure 20 represents the change in methanol concentration versus the permeation time, and the methanol permeability for PVA and PVA/PSSA@CNTs membranes can be determined by calculating the slope of the line. The obtained results exposed that the membrane characteristics significantly improved with the addition of an appropriate ratio of hydrophilic PSSA@CNTs domains, which decreased the permeability of methanol and increased electrochemical selectivity^50,109^. The sorption data is in good accord with the change in the membrane’s methanol permeability. Therefore, the selective sorption of water from a methanol-water mixture is favored by the increased hydrophilic character of the (PVA/1%PSSA@CNTs-CA) membrane. The methanol conducting channel has, therefore, changed from being direct conduct to a circuitous one as a result of the microstate changing. Consequently, the membrane’s methanol permeability dropped. Furthermore, by calculating the efficiency factor(ɸ), which is a ratio of proton conductivity (σ) to methanol permeability, the effectiveness of the membranes produced in this investigation was evaluated. The obtained results are presented in Table 4 and show that (PVA/1%PSSA@CNTs-SA) membrane has the highest efficiency value (1.21 × 10^9^ S·cm⁻³·s ), which is higher than Nafion 117 (2.60 × 10^5^ S·cm⁻³·s), so it can be used as a PEM with high efficiency and low cost than Nafion membrane. Table 5 compares the methanol permeability and proton conductivity between the current study and recent research. All results confirm the significant potential of cross-linked PVA/PSSA@CNTs-based membranes.

**Table 4:** Efficiency factor and Methanol permeability values of the PVA and PVA/PSSA@CNTs-CA, PVA/PSSA@CNTs-SA & PVA/PSSA@CNTs-SC-based poly electrolytic membranes.

Membrane	Methanol permeability (cm^2^ sec^− 1^)	Efficiency factor (S·cm⁻³·s)
Pristine PVA	4.8 ⋅ 10^− 7^	2.79⋅ 10^2^
PVA/1%PSSA@CNTs-CA	2.5 ⋅ 10^− 8^	2.10⋅ 10^6^
PVA/ 1%PSSA@CNTs-SA	4.5 ⋅ 10^− 9^	1.36⋅ 10^7^
PVA/ 1%PSSA@CNTs-SC	3.23⋅ 10^− 9^	1.75⋅ 10^7^
Nafion-117	3.39 ⋅ 10^− 6^	2.6 ⋅ 10^5^

### Comparative discussion with other reported studies

Table 5 compares the results of our developed membranes based on PVA/1%PSSA@CNTs-SC with other reported studies, particularly methanol permeability and proton conductivity. Our membrane demonstrates methanol permeability of (4.5 × 10^− 9^ cm² s⁻¹), substantially lower than Nafion-117 (3.39 × 10⁻⁶ cm² s⁻¹). This improvement stems from the robust hydrogen-bonded structure created by CA, PVA, and SSCA interactions, which hinders methanol crossover effectively. Simultaneously, the PVA/1%PSSA@CNTs-SA membrane achieves a high proton conductivity of (6.12 ± 0.18 × 10^− 2^ S cm⁻¹) close to Nafion-117 (1 × 10⁻² S cm⁻¹) while matching or surpassing values from numerous modified SPEEK- and PVA-based membranes. Unlike PBI@SiNF, PVA/PVP + SSA+PB-M, and Ph-PVA/CA membranes, which have low proton conductivity, our design delivers an optimal balance of ion transport and fuel resistance. This trade-off is essential for use as a PEM, ensuring efficient proton conduction alongside minimal methanol crossover.

**Table 5:** Competitive performance of the developed PEMs compared to reported data.

Membrane	Methanol permeability (cm^2^ sec^− 1^)	Proton conductivity (S cm^− 1^)	References
SPI/SPSGO	4.5 × 10^− 7^	9.62 × 10^− 2^	^110^
SPEEK/Fe3O4@TiO_2_-SO_3_H	2.2 × 10^− 5^	8.1 × 10^− 2^	^111^
SPEEK/PSSA/CNT	5.3 × 10^− 7^	9.5 × 10^− 2^	^39^
SPEEK/MoS2@CNTs	2.7 × 10^− 6^	13.1 × 10^− 2^	^107^
PVA/3%SSCA-MA	2.52 × 10^− 7^	7.8 × 10^− 2^	^55^
PBI@SiNF	-	4 × 10^− 3^	^112^
PVA/PVP + SSA+PB) PB-M(	-	2.39 × 10^− 2^	^113^
Ph-PVA/CA	1.08 × 10^–10^	5 × 10^− 2^	^114^
Nafion 117	3.39 × 10^− 6^	1 × 10⁻²	^115^
PVA/1%PSSA@CNTs-SA	4.5 × 10⁻⁹	(6.12 ± 0.18) x 10^− 2^	This work

## Conclusion

Crosslinked PVA/PSSA@CNTs membranes were successfully prepared using a solution casting procedure. The functional and structural characteristics of the obtained membranes were recognized using different characteristic techniques such as TEM, EDX, SEM CHNS, Raman spectra, and FT-IR spectral analysis. FT-IR shows the achievement of intermolecular bonding occurs among the membrane blend constituents and confirms the functionalization of PVA attainment after using PSSA@CNTs. Furthermore, TEM and SEM illustrate that none of the synthesized PVA/ PSSA@CNTs membranes exhibited incompatibility inside their backbone matrices. In addition, physicochemical features were measured and studied of the prepared membranes using tensile strength, strain (%), methanol uptake, ion exchange capacity, water sorption, contact angle, thermal stability, and membrane efficiency. The characterization results indicated that utilizing PSSA@CNTs at different quantities through the induction of –SO_3_H groups successfully transformed the PVA membrane from a poor proton conductive membrane to a highly electrochemical characteristic. Significantly, chemical cross-linkers used in this research (SA and CA) achieved their objective by water resistance and enhancing the mechanical tensile strength values of the prepared PVA/PSSA@CNTs membranes, enabling them to endure operational circumstances. The IEC values rose as the PSSA@CNTs quantity in the polymer integration increased due to rising sulfonation group capacities. Among the developed membranes, the PVA/1%PSSA@CNTs-SA membrane demonstrated the best overall performance. This evaluation encompassed proton conductivity (6.12 ± 0.18) × 10⁻² S/cm, ion exchange capacity (3.03 meq/g), moderate In-plane swelling (ΔA ≈ 12.87 ± 0.7%), water uptake (45.11%), low methanol uptake (9.37 ± 2%), low methanol permeability (4.5 × 10⁻⁹ cm² s⁻¹), and appropriate tensile strength (104.87 MPa). Taking together, these characteristics promote efficient proton transport while preserving membrane integrity. As a result, this membrane shows superior performance to the commercially available Nafion-117 membrane under similar conditions. Overall, this assessment demonstrates the importance of carefully balancing proton conductivity, swelling, and methanol crossover to optimize the performance of PVA/PSSA@CNTs-based proton exchange membranes. As a result, the developed membrane’s mechanical stability was enhanced by using CA and SA cross-linkers. At the same time, methanol absorption diminished, hence providing effective methanol crossover barrier characteristics. The PVA/PSSA@CNTs membranes eventually display satisfactory electrochemical properties as shown in proton conductivity, IEC, membrane efficiency, thermal stability, and mechanical stability results. So, the prepared membranes based on PVA/PSSA@CNTs are self-extinguished and can act as eco-friendly membrane electrolytes.

## Data Availability

The authors confirm that the data supporting the findings of this study are available within the article.
